# Self-assembled quercetin-zinc supramolecular nanoparticles exhibit strong antibacterial and anti-biofilm activity against methicillin-resistant *Staphylococcus aureus*

**DOI:** 10.3389/fmicb.2026.1908591

**Published:** 2026-09-04

**Authors:** Ying Chen, Xiu-Zhen Chen, Wei-Xiao Wang, Le-Le Xiong, Ming-Yu Jiang, Guang-Ming Zhang, Zi-Xuan Cui, Zhi-Peng Chen, Bai-Ling Zhang, Wei Chen

**Affiliations:** 1 Clinical Research Center, The Second Hospital of Nanjing, Affiliated to Nanjing University of Chinese Medicine, Nanjing, China; 2 Department of Pharmacy, Nanjing University of Chinese Medicine, Nanjing, China; 3 Department of Tuberculosis, The Second Hospital of Nanjing, Affiliated to Nanjing University of Chinese Medicine, Nanjing, China; 4 Department of Blood Transfusion, The Second Affiliated Hospital of Nanchang University, Nanchang, China; 5 Key Laboratory of Resource Biology and Biotechnology in Western China, Ministry of Education, Provincial Key Laboratory of Biotechnology, College of Life Sciences, Northwest University, Xi'an, China

**Keywords:** antimicrobial, bacteremia, methicillin-resistant *Staphylococcus aureus*, nanoparticles, quercetin, zinc ions

## Abstract

Methicillin-resistant *Staphylococcus aureus* (MRSA) infections remain a major public health challenge because of limited therapeutic options and the increasing prevalence of multidrug resistance. In this study, carrier-free quercetin-zinc supramolecular assemblies (Qct-Zn NPs) were fabricated through coordination-associated self-assembly and characterized using TEM, UV-Vis spectroscopy, FTIR, DLS, XPS, and zeta potential analyses. Qct-Zn NPs exhibited potent antibacterial activity against multiple MRSA clinical isolates, with enhanced efficacy compared with quercetin or ZnSO_4_ alone. Time-kill assays demonstrated rapid bactericidal activity, while biofilm assays revealed significant inhibition of biofilm formation and reduction of viable bacteria within mature MRSA biofilms. Mechanistic investigations suggested that Qct-Zn NP treatment was associated with membrane-associated ultrastructural changes, altered intracellular ATP levels, and ROS-associated responses. *In vivo* studies using murine systemic MRSA infection models showed that Qct-Zn NPs reduced bacterial burdens and systemic inflammatory responses while improving survival outcomes. Importantly, antibacterial efficacy was retained when treatment initiation was delayed to 6 h post-infection under the tested experimental conditions. Together with their favorable hemocompatibility and low cytotoxicity, these findings demonstrate that coordination-driven self-assembly of natural flavonoids and biocompatible metal ions represents a potential strategy for developing antimicrobial materials against drug-resistant staphylococcal infections.

## Introduction

Methicillin-resistant Staphylococcus aureus (MRSA) is a major cause of severe bacterial infections, including bacteremia, and is associated with high morbidity and mortality worldwide (Babu Rajendran et al., 2020; Guo et al., 2020; Vestergaard et al., 2019). The increasing prevalence of multidrug-resistant strains has significantly limited the efficacy of conventional antibiotics, highlighting the urgent need for alternative antimicrobial strategies. Quercetin (Qct) is a natural flavonoid widely distributed in plants, fruits, and vegetables (Alizadeh and Ebrahimzadeh, 2022; Yong et al., 2020) and exhibits antioxidant, anti-inflammatory, and antimicrobial activities (Yildirim et al., 2026, 2025b; Tüzün et al., 2026; Yildirim et al., 2025a). However, its clinical application is restricted by poor water solubility and limited bioavailability, which reduces its therapeutic potential (Li et al., 2016). Various approaches have therefore been explored to improve the physicochemical properties and biological activity of quercetin, including formulation strategies and metal ion-associated systems (Riva et al., 2019; Wang H. et al., 2025). Recently, nanomaterial-based antimicrobial platforms have attracted increasing attention due to their tunable physicochemical properties and potential applications against multidrug-resistant pathogens. Metal-based nanoparticles and metal-polyphenol coordination systems have shown promising antibacterial and anti-biofilm activities. However, carrier-based quercetin delivery systems may also face limitations, including insufficient drug-loading efficiency, potential carrier-associated toxicity, and difficulties in precisely controlling or predicting drug release behavior, which may affect their therapeutic performance (Singha Roy et al., 2026; Wang et al., 2016; Tomou et al., 2023). Thus, the development of simple, biocompatible, and effective antimicrobial systems remains highly desirable.

Metal-polyphenol coordination provides a facile strategy for constructing supramolecular nanomaterials by integrating metal ions with polyphenolic compounds under mild conditions (Qin et al., 2023; Jain et al., 2026; Ejima et al., 2013). Among naturally occurring flavonoids, quercetin is particularly suitable for constructing metal-associated assemblies because its catechol and carbonyl-containing structures provide multiple potential coordination sites while retaining intrinsic biological activities, including antimicrobial and anti-inflammatory effects. Therefore, quercetin represents not only a bioactive molecule but also a functional building block for supramolecular material design (Ejima et al., 2013). Quercetin contains multiple hydroxyl and carbonyl groups that can potentially interact with metal ions, which may contribute to altered physicochemical properties and functional performance (Mladěnka et al., 2011; Perron and Brumaghim, 2009). Compared with conventional carrier-based delivery systems, metal-associated quercetin assemblies may reduce the need for additional carrier components while improving structural stability and biological activity (Wang et al., 2016; Jain et al., 2013). Among different metal ions, Zn^2+^ is particularly attractive due to its relatively low toxicity, good biocompatibility, and intrinsic antimicrobial properties. Unlike highly toxic antimicrobial metals, Zn^2+^ is an essential trace element involved in numerous biological processes, providing a favorable balance between antibacterial activity and biosafety (Hajipour et al., 2012). Zn^2+^ has been reported to affect bacterial membrane integrity, interfere with enzymatic processes, and induce oxidative stress-related responses, contributing to antibacterial activity (Yao et al., 2024). These characteristics make Zn^2+^ a promising component for antimicrobial nanoplatform development.

Although quercetin-metal systems and zinc-containing antimicrobial materials have been reported previously, the antibacterial and therapeutic potential of carrier-free quercetin-zinc supramolecular assemblies against multidrug-resistant bacterial infections has not been extensively characterized. In this study, we developed a quercetin-zinc nanoparticle system through coordination-associated self-assembly and evaluated its antibacterial and anti-biofilm activities against MRSA. We further investigated the potential antibacterial mechanisms associated with Qct-Zn NP-mediated bacterial killing and assessed their therapeutic efficacy in a murine MRSA bacteremia model.

## Materials and methods

### Bacterial strains and growth conditions

The bacterial strains used in this study are listed in Supplementary Table 1. *Staphylococcus aureus* strains were obtained from the clinical specimen biobank of the Second Hospital of Nanjing. Antimicrobial susceptibility testing (AST) was performed using the Vitek 2 system, and the susceptibility profiles were presented in Supplementary Table 1. The clinical MRSA isolate 9-11, which exhibited representative susceptibility characteristics among the tested isolates, was selected for subsequent mechanistic, biofilm, and *in vivo* studies. Unless otherwise indicated, *S. aureus* was routinely cultured in Tryptic Soy Broth (TSB) with shaking or on Tryptic Soy Agar (TSA) plates at 37 °C, following standard laboratory procedures for staphylococcal cultivation and antimicrobial susceptibility testing (Balouiri et al., 2016).

### Preparation and characterization of Qct-Zn NPs

Anhydrous zinc sulfate (ZnSO_4_) solution (2 mg/mL) was prepared by dissolving 2 mg of ZnSO_4_ in 1 mL of deionized water. Separately, quercetin solution (2 mg/mL) was prepared by dissolving 2 mg of quercetin in 1 mL of 0.1 M NaOH. The quercetin solution was slowly added dropwise into the ZnSO_4_ solution under continuous gentle agitation at room temperature to ensure uniform dispersion and interaction between quercetin and Zn^2+^ ions. The initial feeding molar ratio of quercetin to Zn^2+^ was approximately 1:2. Subsequently, 8 mL of PBS (1 ×, pH 7.4) was added to the mixture, and the pH was adjusted to 7.4-7.8 using 0.1 M HCl. The mixture was further stirred for 10 min at room temperature. The assembled Qct-Zn NP fraction was collected by centrifugation at 12,000 rpm for 10 min, washed three times with PBS, and resuspended in PBS for subsequent experiments (Cheng et al., 2024; Liu et al., 2020; Wang et al., 2023; Gong et al., 2023). The concentration of Qct-Zn NP suspensions was expressed as the nominal concentration calculated from the total amount of precursor materials used for nanoparticle preparation.

The morphology of the resulting nanoparticles was examined by transmission electron microscopy (TEM). Particle size distribution and polydispersity index (PDI) were measured using dynamic light scattering (DLS). Qct-Zn NP suspensions at different nominal concentrations (4-512 μg/mL) were analyzed after appropriate dilution. The nominal concentration of Qct-Zn NPs was calculated based on the total amount of precursor materials used for nanoparticle preparation. UV-Vis absorption spectra of quercetin, ZnSO_4_, and Qct-Zn NPs were recorded using a UV-Vis spectrophotometer to investigate changes in the optical properties associated with nanoparticle formation.

Fourier transform infrared (FTIR) spectroscopy was performed to investigate functional group changes and chemical environment alterations associated with Qct-Zn NP formation. Samples of quercetin, ZnSO_4_, and Qct-Zn NPs were analyzed using an attenuated total reflection (ATR) accessory. Samples were directly placed onto the ATR crystal without additional KBr pellet preparation. Spectra were collected over the range of 4000–400 cm^−1^ with a resolution of 4 cm^−1^, and 32 scans were accumulated for each measurement. The obtained spectra were processed and analyzed to identify characteristic vibrational changes associated with nanoparticle formation. To ensure comparability, all samples were analyzed at the same concentration (16 μg/mL).

### Determination of zeta potential and colloidal stability

The hydrodynamic diameter, polydispersity index (PDI), and zeta potential of Qct-Zn NPs at concentrations ranging from 4 to 512 μg/mL were determined using a Zetasizer Nano ZS instrument (Malvern Instruments, Worcestershire, UK). All measurements were performed at room temperature in triplicate, and data are presented as mean ± SD.

To evaluate the storage stability of Qct-Zn NPs, freshly prepared nanoparticle suspensions at different concentrations were stored at 4 °C. Particle size and PDI were monitored daily for 7 consecutive days using dynamic light scattering under identical experimental conditions. Measurements were performed in triplicate, and the results were expressed as mean ± SD.

### X-ray photoelectron spectroscopy (XPS) analysis

X-ray photoelectron spectroscopy (XPS) analysis was performed to investigate the surface elemental composition and chemical environments of Qct-Zn NPs. Samples of quercetin, ZnSO_4_, and Qct-Zn NPs were prepared on conductive substrates and analyzed using an XPS instrument (Thermo Scientific K-Alpha) equipped with a monochromatic Al Kα X-ray source.

Survey spectra were collected to identify elemental compositions, and high-resolution spectra of C 1s, O 1s, and Zn 2p regions were acquired for detailed chemical-state analysis. For each sample, five consecutive scans were collected from the same analysis area and accumulated to improve the signal-to-noise ratio.

XPS data were processed using Thermo Scientific Avantage software. The Smart background subtraction model was applied, and peak fitting was performed using a Gaussian–Lorentzian product (GLP) line shape. Binding energies were calibrated using the C 1s peak at 284.8 eV. Quantitative elemental analysis was performed based on integrated peak areas corrected using the relative sensitivity factors (RSFs) provided by the instrument software (C 1s: 1.000, O 1s: 2.881, and Zn 2p: 31.861).

To minimize possible beam-induced chemical alterations, measurements were conducted using a monochromatic Al Kα source without Ar ion sputtering. The analysis conditions were maintained at 12 kV accelerating voltage, 6 mA emission current, and a 400 μm X-ray spot size. No obvious spectral evolution was observed during repeated scans. All XPS measurements were performed on intact samples without sputter-depth profiling to preserve the native chemical environment of the quercetin-n assembly.

### Inhibitory zone assay

The inhibitory zone assay was conducted as previously described (Gong et al., 2023). Briefly, an overnight culture of MRSA was diluted 1:100 into fresh TSB and incubated at 37 °C until reaching the logarithmic phase. Then, 500 μL of bacterial suspension (OD_600_ 0.5) was added to 6 mL of molten TSB (0.8% agar), mixed thoroughly, and poured onto 10 mL TSB solid agar plates. After solidification, sterile round filter paper discs (about 6 mm in diameter) were aseptically placed on the agar surface, and 5 μL of drug at the corresponding concentrations ranging from sub-MIC to multiple-fold MIC concentrations was added to each disc. The plates were inverted and incubated at 37 °C for 24 h. The experiments were performed in triplicate.

### Determination of minimum inhibitory concentration (MIC) of drugs

The minimum inhibitory concentration (MIC) was determined using the standard broth microdilution method according to the Clinical and Laboratory Standards Institute (CLSI) guidelines (M07), as previously described (Awasthi et al., 2026). TSB (100 μL) was dispensed into each well of a 96-well plate, followed by the addition of antimicrobial agents at serial concentrations ranging from 4 to 512 μg/mL.

Quercetin was initially dissolved in DMSO and subsequently diluted in TSB to the desired concentrations. ZnSO_4_ was dissolved in sterile water. For comparison with Qct-Zn NPs, a freshly prepared Qct/ZnSO_4_ mixture was generated by mixing quercetin and ZnSO_4_ solutions immediately before MIC testing at the same precursor molar ratio used for Qct-Zn NP synthesis. Unlike Qct-Zn NP preparation, the Qct/ZnSO_4_ mixture was not subjected to alkaline dissolution, PBS-induced assembly, pH adjustment, or purification procedures. Because direct mixing of Qct and ZnSO_4_ under the MIC assay conditions resulted in visible precipitation, compound-only controls without bacterial inoculation were included to distinguish compound-associated turbidity from bacterial growth.

For Qct-Zn NP testing, nanoparticle-only controls without bacterial inoculation were included to evaluate background absorbance and potential interference caused by nanoparticle-associated scattering or precipitation. Subsequently, 100 μL of bacterial suspension (adjusted to OD_600_ 0.02, approximately 5 × 10^5^ CFU/mL) was added to each well, resulting in a final volume of 200 μL. Growth controls (bacteria without antimicrobial agents), sterility controls (medium without bacteria), and DMSO vehicle controls were included in each experiment. The final DMSO concentration was maintained below 1% (v/v), which showed no detectable effect on bacterial growth. After incubation at 37 °C for 24 h, bacterial growth was evaluated by measuring OD_600_. The MIC was defined as the lowest concentration that completely inhibited visible bacterial growth, as confirmed by OD_600_ measurements. All experiments were performed in triplicate.

### Determination of bacterial growth curve

This experiment was conducted as previously described with minor modifications (Balouiri et al., 2016). TSB (100 μL) was dispensed into each well of a 96-well plate, followed by the test compounds at varying concentrations. Testing concentrations in each assay were selected based on MIC values and preliminary screening results to ensure coverage of relevant inhibitory and sub-inhibitory ranges. Bacterial inoculum (100 μL, OD_600_ 0.02) was then added to each well, yielding a final volume of 200 μL. Positive (drug-free) and negative (bacteria-free) control columns were included. For each treatment, 8 independent replication wells were set up. The plate was immediately placed in a microplate reader, and the OD_600_ was measured automatically every hour for 24 h at 37 °C using independent biological replicates. The experiments were performed in triplicate.

### Time-kill assay

This experiment was conducted as previously described with minor modifications (Wayne, 1999). Overnight cultures of MRSA were diluted 1:100 into fresh TSB and incubated at 37 °C to the logarithmic phase. Bacterial cells were then harvested by centrifugation and washed three times with PBS. The experiment comprised four groups: three treatment groups exposed to Qct-Zn NPs at 1 × MIC, 8 × MIC and 32 × MIC, respectively, and an untreated control group. The selected concentrations (1 ×, 8 ×, and 32 × MIC) were chosen based on preliminary experiments to represent slow, intermediate, and rapid bactericidal effects. Each group was incubated at 37 °C with shaking. At the indicated time points, aliquots were collected, serially diluted, and plated on TSB plates for viable bacterial counting. Following incubation at 37 °C for 24 h, colonies were enumerated, and viable CFU/mL was calculated to construct time-kill curves. The experiments were performed in triplicate.

### Drug inhibition rate assay

The inhibition rate assay was performed according to the protocol previously described (Fu et al., 2024; Huang et al., 2020). TSB (100 μL) was dispensed into each well of a 96-well plate, followed by the test compounds at varying concentrations. Bacterial inoculum (100 μL, OD_600_ 0.02) was added to each well to achieve a final volume of 200 μL. Columns containing growth controls (bacteria without drug) and solvent controls (TSB only) were included. The plate was incubated at 37 °C for 24 h. Subsequently, OD_600_ was measured using a microplate reader. The percentage inhibition of bacterial growth was calculated using the following formula: Inhibition rate (%) = 1-(ODsample-ODsolvent)/(ODblank bacteria-ODsolvent) × 100%. where ODsample represents the drug-treated wells, ODblank bacteria represents the growth control (drug-free bacterial suspension), and ODsolvent represents the medium-only control. To assess bactericidal effects, 5 μL aliquots from wells containing 16 μg/mL of drug were withdrawn, serially diluted, and spotted onto TSB agar plates to observe residual bacterial growth. The experiments were performed in triplicate.

### Confocal laser scanning microscopy (CLSM)

Bacterial viability was assessed by laser confocal microscopy as previously described (Vishwakarma et al., 2021; Wang X. et al., 2025; Cao et al., 2025). Briefly, MRSA was cultured overnight in TSB, diluted to an OD600 of 0.01, and incubated to logarithmic phase. The bacterial suspension was harvested, washed three times with PBS, and divided into four groups (10 mL each). Each group was treated with 200 μL of PBS (control), 128 μg/mL quercetin (Qct), 128 μg/mL ZnSO_4_, or 128 μg/mL Qct-Zn NPs, respectively, followed by incubation at 37 °C for 6 h.

According to the Live/Dead Bacterial Staining Kit instructions, the samples were stained with SYTO-9 (final concentration 7.5 μM) and propidium iodide (PI, final concentration 30 μM) for 30 min at room temperature in the dark. Subsequently, 10 μL of stained suspension was placed onto a clean glass slide precoated with a thin layer of 1% agarose, covered with a coverslip, and gently compressed to distribute bacteria evenly. Excess liquid was absorbed with blotting paper. Imaging was performed using a Zeiss LSM900 confocal microscope. SYTO-9 (excitation: 483 nm; emission: 503 nm) stained viable bacteria, whereas PI (excitation: 493 nm; emission: 636 nm) stained bacteria with compromised membrane integrity.

For semi-quantitative analysis of bacterial membrane damage, CLSM images were further analyzed using ImageJ software. The fluorescence channels were separated, and identical threshold settings were applied to all images within the same experiment. The relative PI-positive fluorescence area was calculated as the ratio of PI-positive area to the total bacterial fluorescence area (SYTO-9-positive area plus PI-positive area). At least five randomly selected fields from each group were analyzed, and three independent experiments were performed.

### Biofilm inhibition and established biofilm reduction assays

The biofilm inhibition assay was performed to evaluate the ability of Qct-Zn NPs to prevent biofilm biomass formation, as previously described (Wang et al., 2022; Piao et al., 2022; Stepanovic et al., 2000). MRSA was cultured in the diluted TSB (66%) supplemented with 0.2% glucose. The bacterial suspension was adjusted to an OD_600_ of 0.02 using the same medium. For the assay, 100 μL of bacterial suspension was added to each well of a 96-well plate containing 100 μL of TSB with varying drug concentrations (final volume: 200 μL per well). Growth controls (bacteria without drug) and medium-only blanks were included. Following incubation at 37 °C for 24 h, the plate was gently washed three times with distilled water to remove non-adherent bacteria and dried for 10 min. The biofilms were stained with 0.1% crystal violet (200 μL per well) at 37 °C for 30 min. Excess dye was removed, and the wells were washed three times with water, dried for 10 min, and then destained with absolute ethanol (200 μL per well) for 30 min. The absorbance was measured at 570 nm (OD_570_) to quantify biofilm biomass. All experiments were performed in triplicate.

The established biofilm reduction assay was conducted as previously described (Wang Y. et al., 2025; Peng et al., 2025). To establish mature biofilms, bacterial suspensions (OD_600_ 0.01) in 66% TSB containing 0.2% glucose were dispensed into a 96-well plate and incubated at 37 °C for 48 h. The culture medium was then aspirated, and adherent biofilms were gently washed once with fresh medium. Subsequently, 200 μL of drug solutions at gradient concentrations was added to the wells, and the plate was incubated at 37 °C for 24 h. After treatment, the liquid was removed, and three random wells per group were selected. The biofilms were scraped and resuspended in 200 μL of PBS by repeated pipetting. The suspensions were transferred to sterile tubes, serially diluted, and plated onto TSB agar for colony enumeration to determine residual viable bacteria (CFU/mL). All experiments were performed in triplicate.

Biofilm was observed following our previous protocol (Wang et al., 2024). MRSA biofilms were cultured in 96-well plates as described above. Following the 48-h formation period, the biofilms were treated with 200 μL of Qct-Zn NPs (32 μg/mL) or left untreated (control) and incubated at 37 °C for 24 h. Subsequently, the wells were excised and fixed overnight at 4 °C in electron microscopy fixative to ensure complete preservation of biofilm architecture. The fixed samples were then dehydrated through a graded ethanol series, critical point dried, sputter-coated with gold, and examined by scanning electron microscopy.

### Morphological observation by SEM and TEM

Morphological changes in MRSA were examined by SEM and TEM. The samples were processed according to the previous protocol (Xu et al., 2020, 2017). Briefly, logarithmic-phase MRSA was resuspended in PBS and incubated with Qct-Zn NPs (128 μg/mL) at 37 °C for 6 h; untreated bacteria served as the control. Following incubation, bacterial cells were harvested by centrifugation and fixed overnight at 4 °C in electron microscopy fixative. The fixed samples were washed three times with PBS and dehydrated through a graded ethanol series (30%, 50%, 70%, 80%, 90%, 95%, and 100%), with three final washes in anhydrous ethanol (10 min each). Samples were then critical point dried using liquid CO_2_ substitution to preserve cellular architecture. Dried specimens were mounted on aluminum stubs using carbon tape, sputter-coated with gold, and examined using a Hitachi S-3000N SEM (Japan). Ultrastructural changes were analyzed by TEM. Dehydrated samples were embedded, sectioned, and stained with uranyl acetate and lead citrate, then placed on copper grids. The grids were inserted into the sample holder and imaged at an accelerating voltage of 80-120 kV. Morphological alterations after Qct-Zn NP exposure were analyzed and compared with untreated controls.

### Determination of extracellular and intracellular ATP levels

To evaluate the effects of Qct-Zn NPs on bacterial energy metabolism, extracellular and intracellular ATP levels were determined using an enhanced ATP assay kit (Beyotime Biotechnology, China; Cat. No. S0027; Lundin, 2000). Briefly, overnight cultures of MRSA strain 9-11 were diluted to an initial OD_600_ of 0.01 in TSB and grown to the exponential phase. Bacterial cells were harvested by centrifugation, washed twice with PBS, and resuspended to an OD_600_ of 0.5. The suspensions were then treated with Qct-Zn NPs (128 μg/mL, 8 × MIC) or Vancomycin (16 μg/mL, 8 × MIC) at 37 °C for 6 h without shaking. Untreated bacteria served as controls. After treatment, bacterial suspensions were centrifuged to collect the supernatants for extracellular ATP determination. Before analysis, bacterial suspensions were adjusted to the same cell density to ensure comparable biomass among groups. The bacterial pellets were then lysed according to the manufacturer's instructions to obtain intracellular extracts for ATP measurement. Luminescence was measured using a microplate reader, and ATP levels were expressed as arbitrary units (AU). Experiments were performed independently at least three times.

### Detection of intracellular ROS-associated fluorescence

Intracellular oxidative stress-associated fluorescence was measured using a Reactive Oxygen Species Assay Kit (Beyotime Biotechnology, China; Cat. No. S0033S) based on the oxidation-sensitive fluorescent probe DCFH-DA (Wang et al., 2018). Briefly, overnight cultures of MRSA strain 9-11 were diluted to an initial OD_600_ of 0.01 in TSB and grown to the exponential phase. After washing twice with PBS, bacterial suspensions were adjusted to an OD_600_ of 0.5 and treated with Qct-Zn NPs (128 μg/mL, 8 × MIC), vancomycin (16 μg/mL, 8 × MIC), or Rosup (50 μg/mL; positive control) at 37 °C for 6 h without shaking. Sterile water was added to the untreated control group. Before analysis, bacterial suspensions were adjusted to the same cell density to ensure comparable biomass among groups. Subsequently, DCFH-DA was added to the bacterial suspensions at a final concentration of 10 μM and incubated at 37 °C for 20 min in the dark. After incubation, the cells were washed three times with PBS to remove excess probe. Fluorescence intensity was measured using a fluorescence microplate reader with excitation and emission wavelengths of 488 and 525 nm, respectively. All experiments were performed independently at least three times.

### Cytotoxicity assay

Cell viability was determined using a CCK-8 kit according to the manufacturer's protocol (Ishiyama et al., 1997). Two mammalian cell lines, normal human hepatocytes (LO2) and human bronchial epithelial cells (BEAS-2B) were seeded at a density of 5 × 10^3^ cells per well in 96-well plates (Wang et al., 2024) and cultured at 37 °C with 5% CO_2_ for 24 h to allow attachment. The culture medium was then replaced with 100 μL of fresh complete medium containing serial dilutions of Qct-Zn NPs. Negative controls consisted of untreated cells, and blank controls consisted of wells containing only culture medium without cells. Following 24 h incubation with the test compounds, 10 μL of CCK-8 solution was added to each well, and the plate was incubated for an additional 2 h at 37 °C protected from light. The absorbance at 450 nm was measured using a microplate reader. Cell viability was calculated relative to untreated control cells. The experiments were performed in triplicate.

### Hemolysis assay

The hemocompatibility of Qct-Zn NPs was evaluated using fresh human erythrocytes. Fresh whole blood from healthy donors was centrifuged at 1500 × g for 10 min to separate plasma and red blood cells (RBCs). The supernatant was discarded, and the RBC pellet was washed three times with PBS (centrifugation at 1500 × g for 10 min each). The washed RBCs were resuspended in PBS to prepare a 4% (v/v) erythrocyte suspension.

Subsequently, serial dilutions of the test compounds and an equal volume of 4% RBC suspension were added to a V-bottom 96-well plate. The plate was incubated at 37 °C for 1 h with gentle agitation, then centrifuged at 1500 × g for 10 min. The supernatant (100 μL) was carefully transferred to a flat-bottom 96-well plate, and the absorbance at 414 nm was measured using a microplate reader. PBS and 0.1% Triton X-100 served as negative (0% hemolysis) and positive (100% hemolysis) controls, respectively (Wang L. et al., 2025). The percentage hemolysis was calculated using the following formula: Hemolysis rate % = [(sample absorbance—negative control absorbance) / (positive control absorbance—negative control absorbance)] × 100%. The experiments were performed in triplicate.

### Evaluation of *in vivo* tolerability of Qct-Zn NPs

This animal protocol was approved by the Institutional Animal Care and Use Committee of Nanjing University of Chinese Medicine and conducted in accordance with institutional guidelines for laboratory animal care. All procedures were performed following the principles of the 3Rs. Mice were anesthetized with isoflurane inhalation before euthanasia by cervical dislocation.

Eight-week-old female Kunming mice were purchased from an accredited laboratory animal center and housed under standard laboratory conditions (room temperature, 50-60% humidity, and a 12 h light/dark cycle). After 1 week of acclimatization, mice were randomly divided into three groups (*n* = 5 per group): (1) PBS control group, (2) Qct-Zn NPs administered by intraperitoneal (IP) injection (37.5 mg/kg), and (3) Qct-Zn NPs administered by intravenous (IV) tail vein injection (37.5 mg/kg). Treatments were administered once daily for six consecutive days. The potential adverse effects of Qct-Zn NPs were evaluated by monitoring body weight and clinical signs throughout the observation period. A standardized clinical scoring system was used to assess the general health status of mice after treatment (Shrum et al., 2014): 0, normal appearance and behavior; 1, mild piloerection and partial eye closure with strong avoidance response; 2, moderate piloerection, reduced mobility, eye discharge, or delayed response; 3, minimal response to stimuli; 4, no response to touch with closed eyes; and 5, death.

At day 7, blood samples were collected for hematological analysis, including white blood cell and neutrophil counts. Serum inflammatory cytokines, including TNF-α, IL-6, and IL-1β, were measured using ELISA kits according to the manufacturer's instructions.

### Establishment of a murine systemic MRSA infection model

Mice were intraperitoneally inoculated with *S. aureus* 9-11 at three different bacterial doses (*n* = 6 per group) to establish a systemic infection model. Group 1 received 1 × 10^7^ CFU per mouse, Group 2 received 5 × 10^7^ CFU per mouse, and Group 3 received 1 × 10^8^ CFU per mouse. Clinical signs and survival were monitored at regular intervals after infection, and survival curves were generated using the Kaplan-Meier method. The observation period was seven days.

### Therapeutic evaluation of Qct-Zn NPs in a murine systemic MRSA infection model

Eight-week-old female Kunming mice were housed under standard laboratory conditions and acclimatized for 1 week before experiments. Animals were weighed and randomly assigned into seven groups (*n* = 12 per group): (1) uninfected control treated with PBS; (2) MRSA-infected control treated with PBS; (3) quercetin (Qct, 7.5 mg/kg); (4) zinc sulfate (ZnSO_4_, 3.75 mg/kg), corresponding to the theoretical Zn^2+^ amount introduced into Qct-Zn NPs at the tested dose based on the precursor feeding ratio; (5) Qct-Zn NPs administered by intraperitoneal injection (IP, 7.5 mg/kg); (6) Qct-Zn NPs administered by intravenous injection (IV, 7.5 mg/kg); and (7) Qct-Zn NPs administered by repeated IP injection (7.5 mg/kg daily for six consecutive days). For the Qct treatment group, quercetin powder was initially dissolved in dimethyl sulfoxide (DMSO) and further diluted with sterile PBS to obtain a final formulation containing 10% DMSO before administration. The final DMSO concentration was selected based on previous reports and was well tolerated in mice. The selected dose of Qct-Zn NPs was determined based on preliminary biosafety evaluation and previous studies investigating quercetin- or zinc-based antibacterial formulations (Jing et al., 2022). Vancomycin (30 mg/kg) was included as a positive control based on previously reported murine MRSA infection models (Shin et al., 2011). The selected dose was within the range commonly used for evaluating Vancomycin efficacy in murine MRSA infection models.

Systemic MRSA infection was induced by intraperitoneal inoculation of *S. aureus* 9-11 (1 × 10^8^ CFU per mouse in 200 μL PBS). Treatments were initiated 1 h post-infection (POI). Two experimental endpoints were predefined. At the early endpoint (6 h POI), four mice per group were euthanized, and blood and major organs were collected for bacterial burden determination by serial dilution and colony counting. Serum samples were collected for inflammatory cytokine analysis, including IL-1β, IL-6, and TNF-α, using commercial ELISA kits according to the manufacturer's instructions. For survival evaluation, the remaining mice were monitored for seven days after infection. Body weight and clinical signs were recorded every 24 h, and survival was analyzed using the Kaplan-Meier method.

### Evaluation of delayed treatment efficacy

To further evaluate the therapeutic efficacy of delayed administration of Qct-Zn NPs, an additional delayed-treatment experiment was performed using the murine systemic MRSA infection model. Mice were intraperitoneally inoculated with 1 × 10^8^ CFU of MRSA strain 9-11 in 0.2 mL PBS and randomly assigned to five groups (*n* = 5 per group): (1) uninfected control; (2) infected PBS-treated control; (3) Qct-Zn NPs treatment initiated at 1 h post-infection; (4) Qct-Zn NPs treatment initiated at 6 h post-infection; and (5) Vancomycin treatment initiated at 6 h post-infection. Qct-Zn NPs were administered intraperitoneally at a dose of 7.5 mg/kg, whereas Vancomycin was administered intraperitoneally at 30 mg/kg. Treatments were administered once daily for six consecutive days.

Mice were monitored for clinical signs and body weight changes at 24 h intervals throughout the 7-day observation period. Clinical scores were assessed according to the criteria described above. Survival was recorded daily and analyzed using the Kaplan-Meier method with GraphPad Prism (version 10.5.0). At the study endpoint, surviving mice were euthanized for downstream hematological and biochemical analyses.

### Histopathological examination

For histopathological analysis, the sections of livers, spleens and kidneys were fixed in 4% paraformaldehyde, embedded in paraffin and stained with hematoxylin-eosin.

### Statistical Analysis

All statistical analyses were performed using GraphPad Prism version 10.5.0. Data are presented as mean ± SD unless otherwise indicated. Differences between two groups were analyzed using unpaired Student's *t*-test. Comparisons among multiple groups were analyzed using one-way or two-way ANOVA followed by Tukey's multiple-comparison test, as appropriate, after verification of normality and homogeneity of variance. Time-dependent datasets, including bacterial growth curves and time-kill assays, were analyzed using two-way ANOVA with treatment and time as factors. CFU values were log_10_-transformed before statistical analysis. Survival curves were analyzed using the Kaplan-Meier method with the log-rank test. A *P* value < 0.05 was considered statistically significant.

## Results

### Self-assembly and physicochemical characterization of Qct-Zn nanoparticles

**Figure 1:**
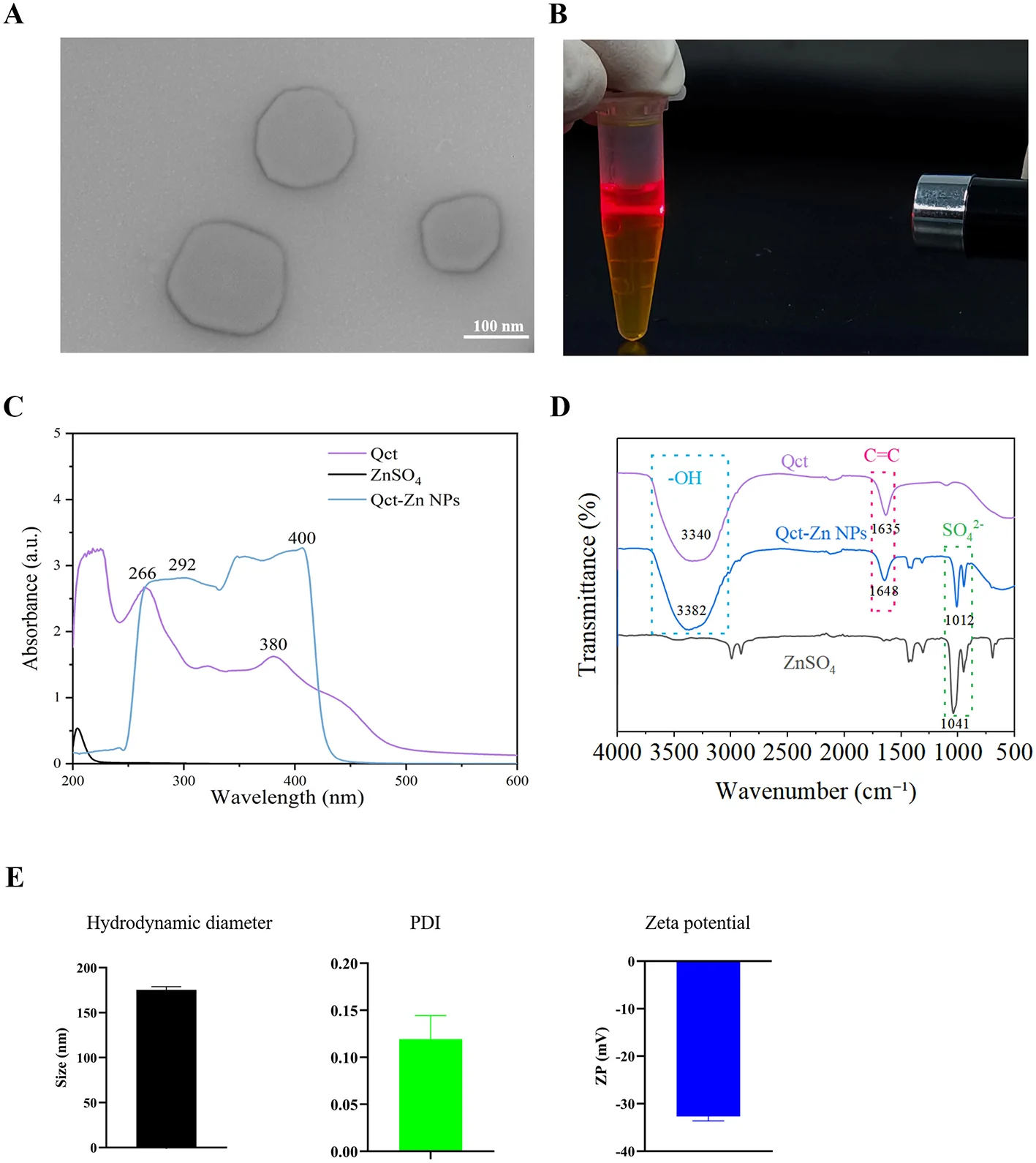
Fabrication and physicochemical characterization of Qct-Zn NPs. **(A)** Representative TEM image showing the spherical morphology of Qct-Zn NPs. Scale bar, 100 nm. **(B)** Tyndall effect of Qct-Zn NPs dispersion under laser irradiation, indicating the formation of a stable colloidal system. **(C)** UV-Vis absorption spectra of quercetin (Qct), ZnSO_4_, and Qct-Zn NPs. **(D)** FTIR spectra of Qct, ZnSO_4_, and Qct-Zn NPs showing changes in oxygen-containing functional groups after nanoparticle formation. **(E)** Hydrodynamic diameter, polydispersity index (PDI), and zeta potential of Qct-Zn NPs measured at a representative concentration of 64 μg/mL. Data are presented as mean ± SD (*n* = 3).

Transmission electron microscopy (TEM) revealed that quercetin (Qct) and zinc-containing species assembled into nanoscale structures with predominantly spherical morphology (Figure 1A). Additional TEM imaging under a wider field of view further confirmed the formation of nanoscale assemblies (Supplementary Figure 1A). The formation of a dispersed colloidal system was further indicated by the Tyndall effect observed for Qct-Zn NPs (Figure 1B). The optical properties of Qct, ZnSO_4_, and Qct-Zn NPs were subsequently analyzed using UV-Vis absorption spectroscopy (Figure 1C). Compared with free Qct, Qct-Zn NPs exhibited red shifts of characteristic absorption bands from 380 to 400 nm and from 266 to 292 nm, accompanied by peak broadening and increased absorbance. These spectral changes indicate alterations in the electronic environment of Qct during nanoparticle formation in the presence of Zn^2+^. Because the absorption features of quercetin are sensitive to the surrounding chemical environment, solvent conditions, and molecular interactions, the observed changes were interpreted as spectral alterations associated with nanoparticle formation rather than definitive evidence of a specific Zn-Qct coordination structure.

Further structural characterization was performed using Fourier-transform infrared (FTIR) spectroscopy (Figure 1D). Several characteristic vibrational regions of Qct exhibited changes after nanoparticle formation. Specifically, the O-H stretching band shifted from 3340 to 3382 cm^−1^, while the absorption region around 1,620–1,650 cm^−1^, containing overlapping contributions from aromatic C=C stretching and chromone C=O stretching vibrations, shifted from 1635 to 1648 cm^−1^. In addition, the absorption region around 1,012–1,041 cm^−1^, containing contributions from sulfate-related vibrations of ZnSO_4_ and C-O-C vibrations of Qct, showed a shift after nanoparticle formation. These spectral changes indicate alterations in the local chemical environment during nanoparticle assembly and are consistent with Zn-associated interactions involving oxygen-containing functional groups of Qct (Kasprzak et al., 2015; Nakamura et al., 2023).

To further characterize the colloidal properties of Qct-Zn NPs, the hydrodynamic diameter, PDI, and zeta potential were determined at a representative concentration of 64 μg/mL (Figure 1E). Qct-Zn NPs exhibited an average hydrodynamic diameter of approximately 175 nm with a low PDI value, indicating a relatively homogeneous particle-size distribution. The zeta potential was approximately −33 mV, indicating a negatively charged nanoparticle surface and favorable electrostatic repulsion under the tested conditions. The observed negative surface potential may originate from multiple surface-associated species, including deprotonated oxygen-containing groups of quercetin and sulfate-related species derived from ZnSO_4_. However, the relative contribution of these components cannot be quantitatively determined based on the current measurements. The concentration-dependent changes in particle size and PDI were further evaluated across concentrations ranging from 4 to 512 μg/mL. As shown in Supplementary Figure 1B, the hydrodynamic diameter ranged from approximately 175 to 309 nm, while PDI values remained below 0.25 across all tested concentrations. The smallest particle size and lowest PDI value were observed at 64 μg/mL. Storage stability was further evaluated by monitoring particle size and PDI during storage at 4 °C for 7 days. No appreciable changes in either parameter were observed during the storage period (Supplementary Figure 1C), suggesting favorable short-term colloidal stability under the tested storage conditions. The concentration-dependent zeta potential changes were further evaluated and are presented in Supplementary Figure 2. Qct-Zn NPs exhibited negative surface charges ranging from approximately −22 to −34 mV. At concentrations between 16 and 512 μg/mL, the absolute zeta potential approached or exceeded 30 mV, suggesting favorable electrostatic repulsion and colloidal stability over the tested concentration range.

Collectively, these characterization results indicate the formation of Qct-Zn nanoparticles with well-defined nanoscale morphology, favorable colloidal properties, and physicochemical characteristics suitable for subsequent antibacterial and biological evaluations.

### XPS analysis reveals Zn-associated chemical environment changes during Qct-Zn NP formation

**Figure 2:**
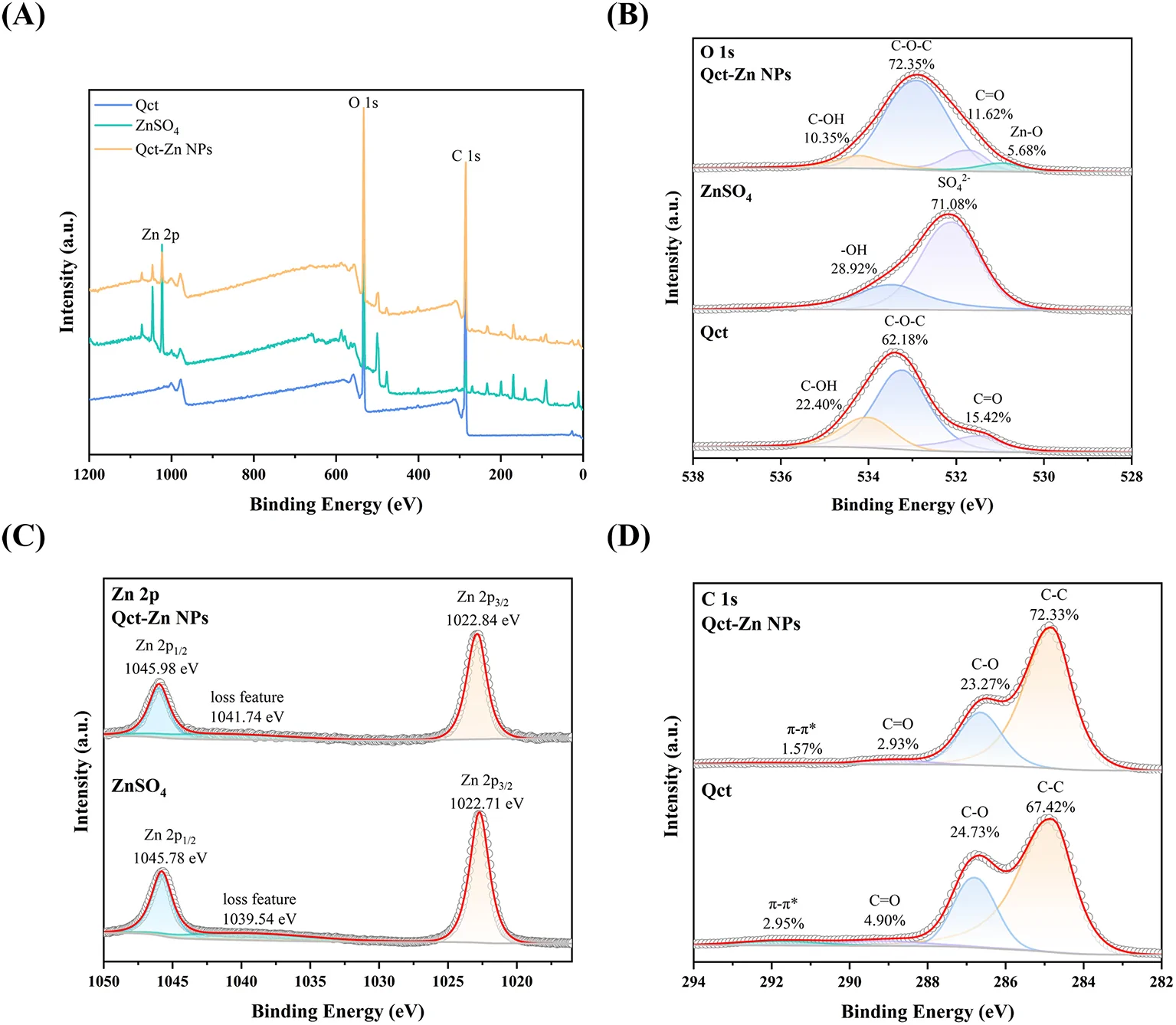
XPS analysis of Qct, ZnSO_4_, and Qct-Zn NPs. **(A)** Survey spectra confirming the elemental composition of the samples. **(B)** High-resolution O 1s spectra showing the emergence of Zn-O coordination signals in Qct-Zn NPs. **(C)** Zn 2p spectra of ZnSO_4_ and Qct-Zn NPs, indicating binding energy shifts after coordination. **(D)** High-resolution C 1s spectra demonstrating changes in carbon chemical states upon nanoparticle formation. These results collectively support the involvement of interactions between Zn^2+^ and oxygen-containing functional groups of quercetin, leading to the formation of stable Qct-Zn nanoparticles.

To further investigate chemical environment changes associated with nanoparticle formation, X-ray photoelectron spectroscopy (XPS) analysis was performed on Qct, ZnSO_4_, and Qct-Zn NPs (Figure 2). The survey spectra confirmed the presence of C, O, and Zn elements in Qct-Zn NPs, indicating the incorporation of Zn-containing species into the quercetin-based nanoparticle system.

High-resolution O 1s spectra revealed differences in oxygen-containing chemical environments among the samples. Qct exhibited characteristic oxygen components associated with C=O, C-O-C, and C-OH species, whereas ZnSO_4_ showed oxygen signals mainly attributed to sulfate species. These oxygen-containing functional groups have been reported to participate in metal-polyphenol interactions, providing potential sites for association with metal ions (Perron and Brumaghim, 2009). Compared with the individual components, Qct-Zn NPs displayed an additional O 1s component at 530.93 eV, which may reflect changes in Zn-associated oxygen environments after nanoparticle formation. In addition, the relative contribution of C-OH species decreased after nanoparticle formation, suggesting that oxygen-containing functional groups of quercetin may participate in interactions with Zn-containing species during nanoparticle assembly. Because sulfate-related oxygen signals partially overlap with oxygen-containing functional groups in the nanoparticle system, the O 1s spectra were interpreted as reflecting overall chemical environment changes rather than direct evidence for a specific coordination structure.

Consistently, the Zn 2p_3/2_ and Zn 2p_1/2_ peaks of Qct-Zn NPs exhibited slight shifts relative to ZnSO_4_, suggesting subtle changes in the local chemical environment of Zn species following nanoparticle formation. C 1s spectra also showed changes in peak distribution and relative intensity after nanoparticle formation, further supporting changes in the chemical environment of carbon-containing components after nanoparticle formation. Considering the relatively small Zn 2p binding-energy shift, these changes were interpreted together with the O 1s, C 1s, FTIR, and UV-Vis results rather than as independent evidence of Zn-quercetin coordination.

RSF-corrected quantitative analysis further revealed the surface elemental composition of Qct-Zn NPs, with Zn, O, and C accounting for 2.20%, 30.43%, and 67.37%, respectively. Because XPS is inherently surface-sensitive, providing information mainly from the outermost surface region of materials (Beamson and Briggs, 1993), these elemental percentages represent the surface composition of the nanoparticles and should not be interpreted as the overall Zn-to-quercetin stoichiometric ratio of the supramolecular assembly.

Collectively, the coordinated changes observed in the O 1s, Zn 2p, and C 1s spectra, together with complementary FTIR and UV-Vis analyses, indicate altered chemical environments during Qct-Zn NP formation. These findings support the involvement of Zn-containing species and oxygen-containing functional groups of quercetin in nanoparticle assembly. However, the specific coordination geometry, binding sites, and molecular-level structure cannot be determined based solely on the current XPS evidence.

### *In vitro* antibacterial activity of Qct-Zn nanoparticles

**Figure 3:**
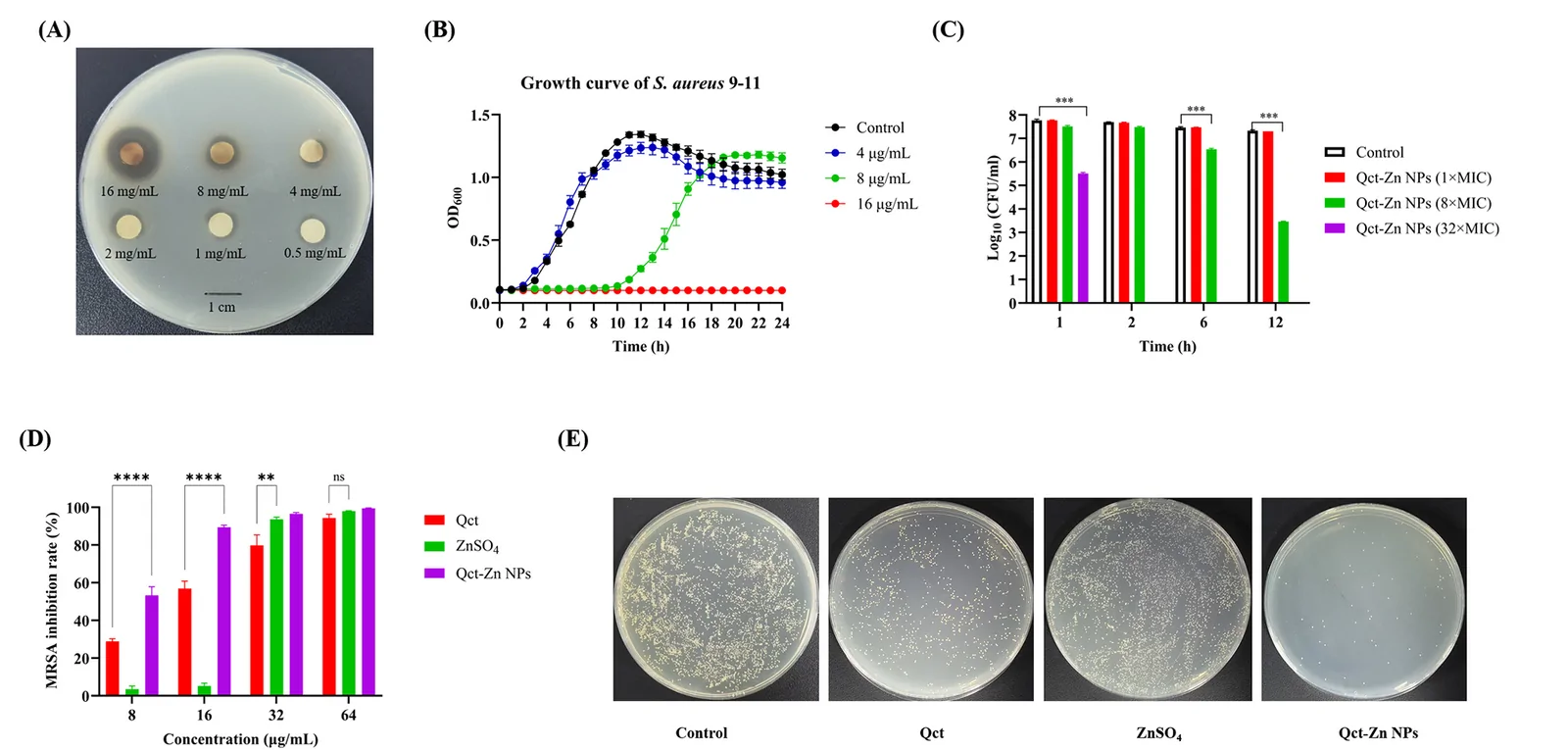
*In vitro* antibacterial activity of Qct-Zn nanoparticles against MRSA 9-11. **(A)** Representative inhibition zones produced by Qct-Zn NPs on MRSA-inoculated agar plates. **(B)** Bacterial growth curves of MRSA following treatment with Qct-Zn NPs at the indicated concentrations. **(C)** Time-kill kinetics showing viable colony-forming units (CFU) over 12 h after exposure to Qct-Zn NPs at 1 ×, 8 ×, and 32 × MIC, demonstrating rapid and concentration-dependent bactericidal activity. **(D)** Inhibition rates of quercetin (Qct), ZnSO_4_, and Qct-Zn NPs against MRSA. **(E)** Representative agar plates from the inhibition assay at 16 μg/mL. Data are presented as mean ± SD. Statistical significance was determined using Student's *t*-test. **p < 0.01; ***p < 0.001; ****p < 0.0001.

A filter paper diffusion assay was performed as a preliminary qualitative assessment of the diffusion-associated antibacterial activity of Qct-Zn NPs. Drug-impregnated filter papers produced clear and well-defined inhibition zones on *S. aureus*-inoculated agar plates, demonstrating effective bacterial growth suppression. The inhibition diameter increased in a dose-dependent manner (Figure 3A). Broth microdilution assays further showed that Qct-Zn NPs had a minimum inhibitory concentration (MIC) of 16 μg/mL against methicillin-resistant *S. aureus* (MRSA) strain 9-11, which was lower than that of either ZnSO_4_ (32 μg/mL) or Qct alone (64 μg/mL), indicating that Qct-Zn NPs exhibited stronger inhibitory activity than the individual components under the tested conditions. To further evaluate whether the enhanced antibacterial activity of Qct-Zn NPs was associated with nanoparticle assembly rather than simple mixing of individual components, a Qct/ZnSO_4_ mixture control was evaluated. Direct mixing of Qct and ZnSO_4_ under MIC assay conditions resulted in visible precipitation. The Qct/ZnSO_4_ mixture exhibited an MIC of 128 μg/mL, whereas Qct-Zn NPs showed an MIC of 16 μg/mL (Supplementary Table 1), suggesting that the pre-assembled Qct-Zn NP formulation exhibited greater antibacterial activity than the directly mixed Qct/ZnSO_4_ preparation under the tested conditions.

To evaluate the generalizability of the antibacterial activity of Qct-Zn NPs, additional clinical *S. aureus* isolates were tested, including eight MRSA strains and one methicillin-susceptible *S. aureus* (MSSA) strain. As summarized in Supplementary Table 2, Qct-Zn NPs exhibited consistent antibacterial activity against all tested isolates. The MIC values were 16 μg/mL for all tested isolates. The MBC values varied among strains, with some isolates exhibiting MBC values of ≤ 128 μg/mL, whereas others showed MBC values exceeding 128 μg/mL. The antibacterial activity of Qct-Zn NPs was observed across isolates with different antimicrobial resistance profiles, suggesting that the activity was not limited to a single MRSA strain.

Growth curve analysis revealed that 16 μg/mL Qct-Zn NPs completely inhibited bacterial proliferation over 24 h, whereas 8 μg/mL Qct-Zn NPs maintained growth suppression during the initial 10 h (Figure 3B). Time-kill assays demonstrated rapid bactericidal activity. Concentrations of 1 ×, 8 ×, and 32 × MIC were selected to represent inhibitory, intermediate, and rapid bactericidal conditions, respectively. Treatment with 8 × MIC eliminated >90% of bacteria within 6 h and achieved a >4-log reduction within 12 h, while 32 × MIC eradicated approximately 1 × 10^8^ CFU within 2 h, indicating marked killing efficacy (Figure 3C). These results indicate rapid and concentration-dependent antibacterial effects of Qct-Zn NPs under the tested conditions.

Consistently, inhibition rate measurements showed that Qct-Zn NPs displayed stronger antibacterial effects than Qct or ZnSO_4_ alone (Figures 3D, E). Collectively, these findings demonstrate that Qct-Zn NPs exhibit potent concentration-dependent antibacterial activity against *S. aureus*, with increased activity compared with the individual components.

### Assessment of MRSA membrane damage induced by Qct-Zn NPs using CLSM

**Figure 4:**
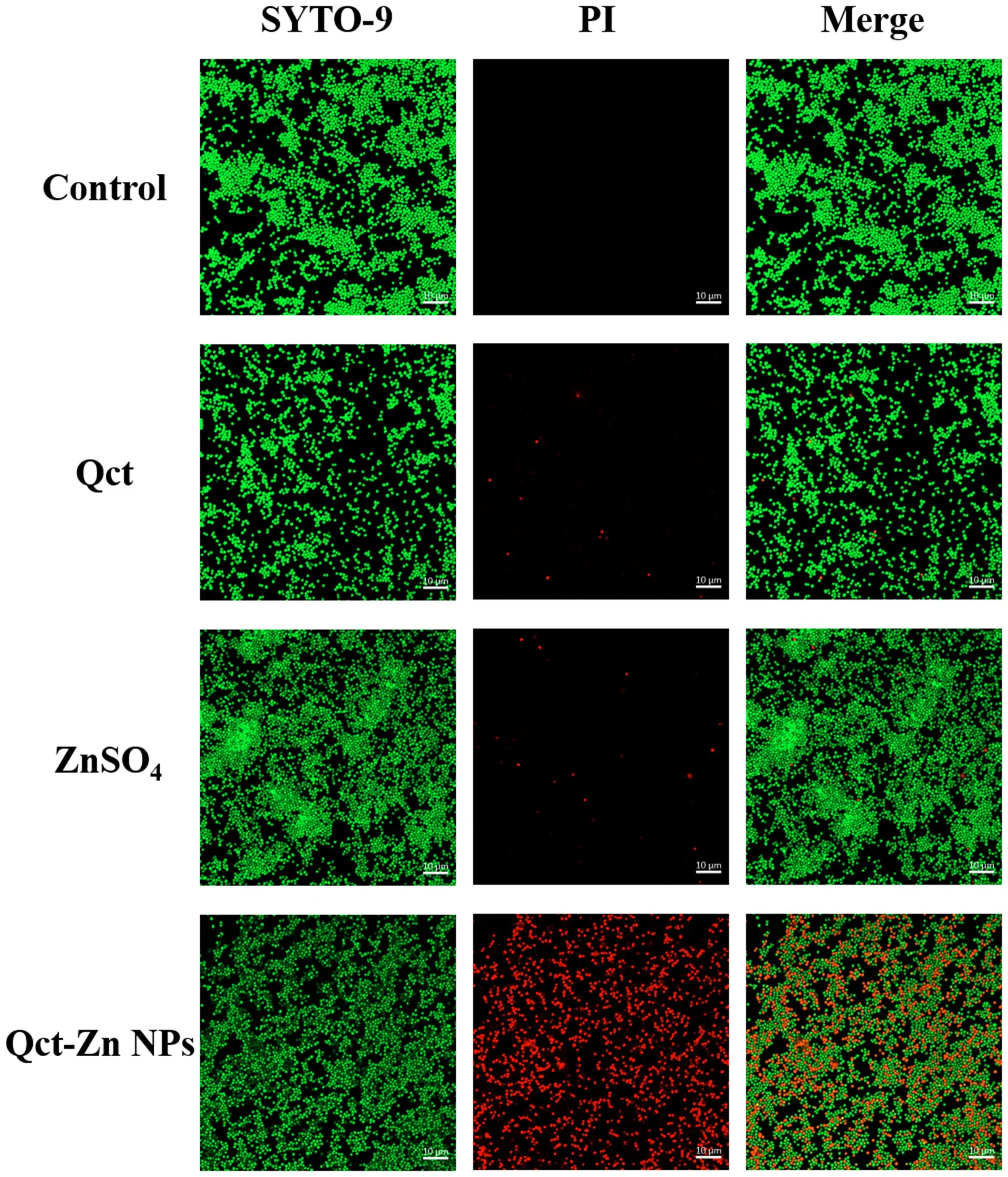
Assessment of bacterial membrane integrity following treatment with Qct-Zn nanoparticles. Log-phase MRSA 9-11 cells were treated with quercetin (Qct), ZnSO_4_, or Qct-Zn NPs (128 μg/mL) for 6 h and stained with SYTO-9 and propidium iodide (PI). Live bacteria with intact membranes were stained green by SYTO-9, whereas bacteria with compromised membrane integrity were stained red by PI. Representative CLSM images are shown. Scale bar: 10 μm.

To further investigate the antibacterial effects of Qct-Zn NPs, bacterial membrane integrity was examined using SYTO-9/PI dual staining coupled with confocal laser scanning microscopy (CLSM). Untreated MRSA cells exhibited predominantly green fluorescence, indicating intact membrane structures and high bacterial viability (Figure 4). In contrast, treatment with Qct or ZnSO_4_ alone resulted in limited PI-positive staining, suggesting partial membrane damage. Notably, MRSA cells treated with Qct-Zn NPs displayed substantially increased red fluorescence and a markedly higher proportion of PI-positive signals compared with individual components. Semi-quantitative analysis of fluorescence signals using ImageJ revealed that the relative PI-positive fluorescence area accounted for approximately 2.0%, 2.4%, and 38.0% of the total bacterial fluorescence area in the Qct, ZnSO_4_, and Qct-Zn NP groups, respectively. These findings suggest that the improved antibacterial activity of Qct-Zn NPs is associated, at least partially, with enhanced bacterial membrane damage.

### Inhibition and reduction of established *S. aureus* biofilms by Qct-Zn nanoparticles

**Figure 5:**
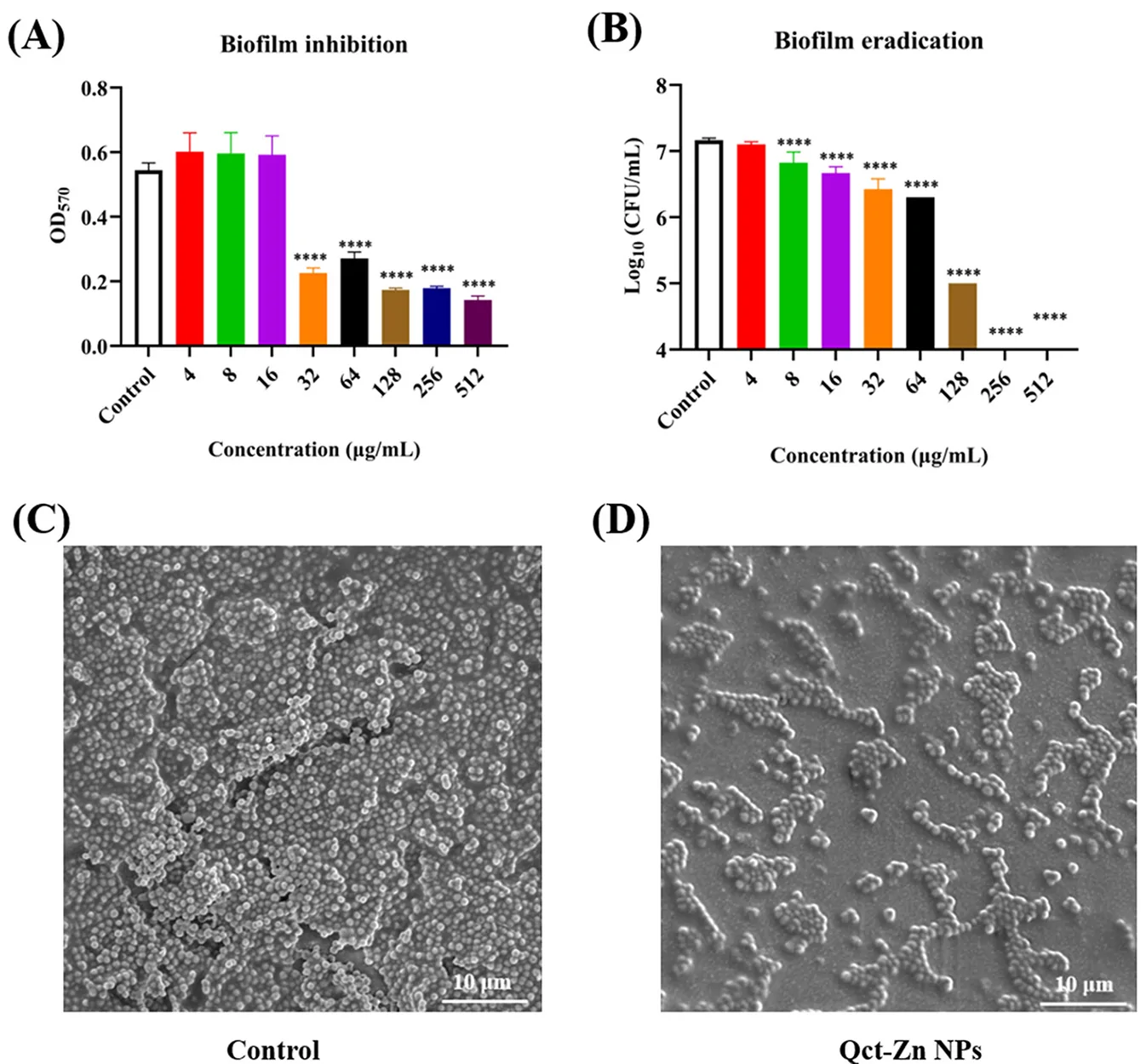
Inhibition of biofilm formation and reduction of established biofilms by Qct-Zn NPs. **(A)** Inhibition of biofilm formation by different concentrations of Qct-Zn NPs evaluated using the crystal violet staining assay. **(B)** Reduction of viable biofilm-associated bacteria after treatment with different concentrations of Qct-Zn NPs determined by colony counting assay. **(C)** Representative SEM images of untreated MRSA biofilms. **(D)** Representative SEM images of MRSA biofilms treated with Qct-Zn NPs. Biofilms were treated with Qct-Zn NPs under the indicated conditions, followed by SEM observation. Scale bar: 10 μm. Statistical significance was analyzed using Student's *t*-test. ****P < 0.0001.

To evaluate the anti-biofilm activity of Qct-Zn NPs, we first assessed their ability to inhibit biofilm formation by *S. aureus* 9-11. Treatment with Qct-Zn NPs markedly reduced biofilm development, with substantial inhibition observed at 32 μg/mL (2 × MIC; Figure 5A). We next evaluated the effects of Qct-Zn NPs on established biofilms. Two-day-old mature biofilms were formed in PVC 96-well plates and quantified by CFU enumeration. Qct-Zn NPs reduced the viability of bacteria associated with established biofilms in a concentration-dependent manner. Treatment with 128 μg/mL (8 × MIC) resulted in a >2-log reduction in viable bacteria within 24 h, whereas 256 μg/mL (16 × MIC) resulted in a further reduction in viable bacteria within established biofilms (Figure 5B).

Scanning electron microscopy (SEM) further supported these observations. Untreated *S. aureus* formed dense and organized biofilm structures on the PVC surface, whereas Qct-Zn NP treatment resulted in fewer and more sparsely distributed bacterial cells and altered biofilm-associated morphology (Figures 5C, D).

Taken together, these results indicate that Qct-Zn NPs inhibit biofilm formation and reduce the viability of bacteria within established biofilms, suggesting their potential application as an anti-biofilm agent against *S. aureus*.

### Ultrastructural analysis of membrane alterations by Qct-Zn nanoparticles

**Figure 6:**
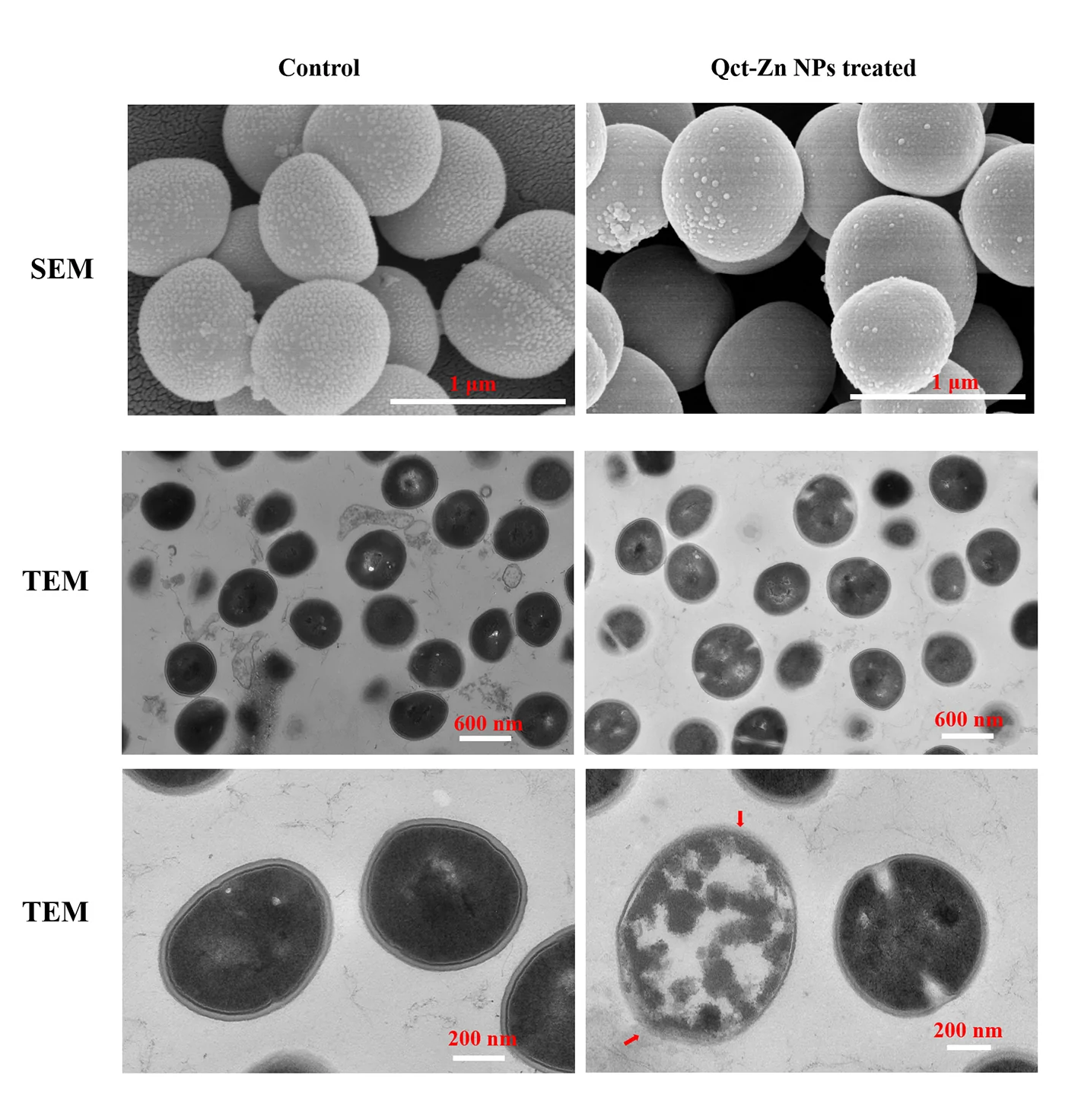
SEM and TEM images of MRSA 9-11 treated with or without Qct-Zn NPs. SEM scale bar: 1 μm. TEM scale bar: 600 nm/200 nm.

Scanning electron microscopy (SEM) showed that Qct-Zn NP-treated bacteria did not exhibit obvious cell rupture or gross structural collapse. Instead, the cell surfaces appeared smoother, with fewer particulate attachments compared with the untreated controls (Figure 6). Transmission electron microscopy (TEM) provided further insight into ultrastructural changes. Control *S. aureus* cells displayed intact membranes and electron-dense cytoplasmic contents, appearing uniformly dark. In contrast, cells treated with Qct-Zn NPs exhibited reduced electron density, increased intracellular vacuolation, and morphological features associated with altered cytoplasmic organization. Higher-magnification images revealed changes in membrane-associated structures and cellular integrity (Figure 6). These observations suggest that Qct-Zn NP treatment is associated with ultrastructural alterations of bacterial cells, including changes in membrane-associated morphology and intracellular organization.

### Qct-Zn NPs alter intracellular ATP levels and ROS-associated fluorescence in MRSA

**Figure 7:**
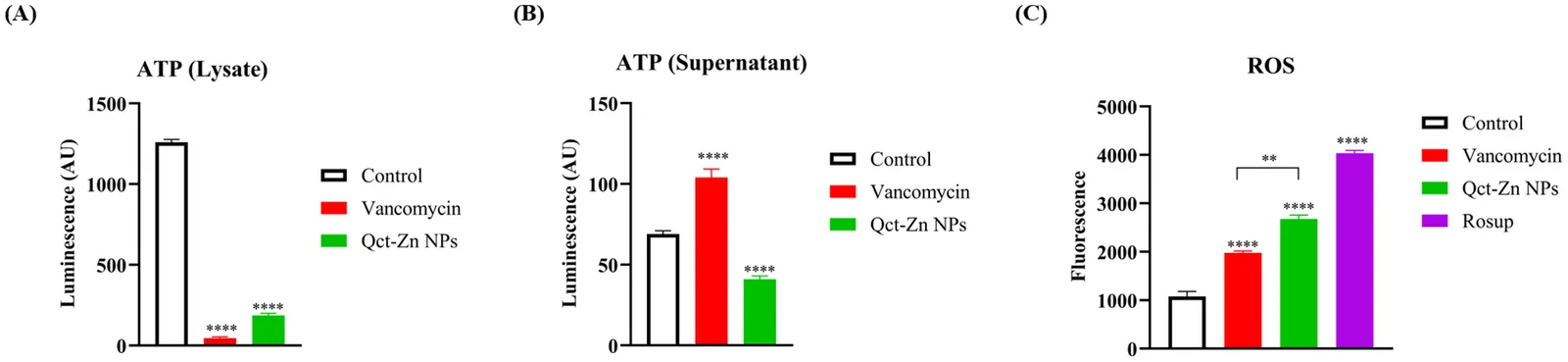
Effects of Qct-Zn NPs on ATP homeostasis and ROS-associated fluorescence changes in MRSA 9-11. Exponential-phase MRSA 9-11 cells were treated with Qct-Zn NPs (128 μg/mL, 8 × MIC) or Vancomycin (16 μg/mL, 8 × MIC) for 6 h at 37 °C. Untreated cells served as controls. **(A)** Intracellular ATP levels measured in bacterial lysates. **(B)** Extracellular ATP levels measured in culture supernatants. **(C)** Intracellular ROS accumulation detected using the DCFH-DA fluorescent probe. Rosup (50 μg/mL) was used as a positive control for ROS induction. ATP levels were determined using a luminescence-based ATP assay kit and expressed as arbitrary units (AU). ROS-associated fluorescence intensity was measured at excitation/emission wavelengths of 488/525 nm. Data are presented as mean ± SD from three independent experiments. Statistical analysis was performed using one-way ANOVA followed by Tukey's multiple-comparison test. ****P < 0.0001 vs. untreated control. A two-tailed Student's *t*-test was used for comparison between Qct-Zn NP and vancomycin-treated groups (**P < 0.01).

To further investigate cellular responses associated with Qct-Zn NP treatment, intracellular ATP levels, extracellular ATP release, and ROS-associated fluorescence were evaluated following treatment with Qct-Zn NPs at 8 × MIC for 6 h. Compared with untreated controls, both vancomycin and Qct-Zn NPs markedly reduced intracellular ATP levels, indicating altered intracellular energy status following antibacterial treatment (Figure 7A). Vancomycin treatment was associated with increased extracellular ATP levels, whereas Qct-Zn NP-treated bacteria did not show a significant increase in extracellular ATP release (Figure 7B).

In addition, both treatments increased intracellular ROS-associated fluorescence compared with untreated bacteria, with Qct-Zn NPs showing higher fluorescence intensity than vancomycin-treated cells (Figure 7C). These results indicate that Qct-Zn NP treatment is associated with changes in intracellular ATP levels and ROS-related fluorescence signals under the tested conditions.

### *In vitro* cytotoxicity and hemocompatibility evaluation of Qct-Zn nanoparticles

**Figure 8:**
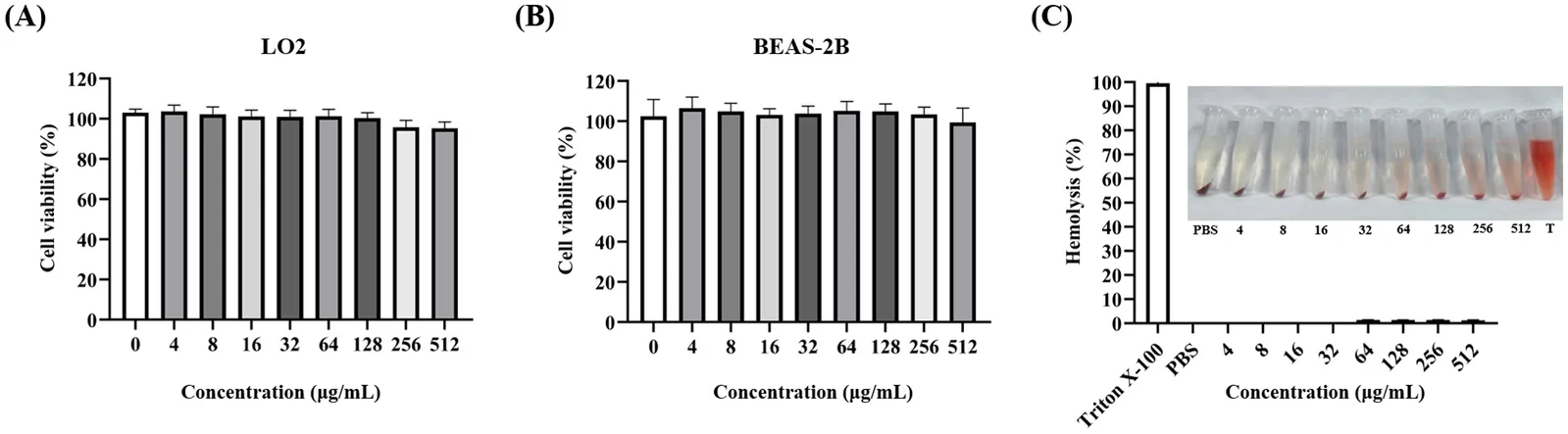
*In vitro* cytotoxicity and hemocompatibility evaluation of Qct-Zn NPs. **(A)** The cytotoxicity of Qct-Zn NPs was evaluated by measuring the cell viability of L02 cells via the CCK8 assay. **(B)** The cytotoxicity of Qct-Zn NPs was evaluated by measuring the cell viability of BEAS-2B cells via the CCK8 assay. **(C)** Hemolytic activity was determined by measuring the release of hemoglobin from human erythrocytes exposed to varying concentrations of Qct-Zn NPs, with PBS and 0.1% Triton X-100 as negative and positive controls, respectively.

As an antimicrobial candidate, the preliminary biosafety profile of Qct-Zn NPs was evaluated. Two mammalian cell lines, normal human hepatocytes (LO2) and human bronchial epithelial cells (BEAS-2B), were selected to assess cytotoxicity. CCK-8 assays showed that Qct-Zn NPs exhibited limited cytotoxic effects toward both cell types, with high cell viability maintained at concentrations up to 512 μg/mL (32 × MIC; Figures 8A, B).

Hemocompatibility was further evaluated using a red blood cell (RBC) hemolysis assay. Qct-Zn NPs induced minimal hemolysis, with less than 2% hemolysis observed at 512 μg/mL (Figure 8C). According to commonly used hemolysis evaluation criteria, hemolysis below 5% is generally considered acceptable, indicating low hemolytic activity under the tested conditions.

The antibacterial activity of Qct-Zn NPs was observed at 16 μg/mL against *S. aureus*, whereas substantially higher concentrations were required to affect mammalian cell viability under the tested conditions. These findings indicate that Qct-Zn NPs exhibited favorable preliminary *in vitro* selectivity and low hemolytic activity within the evaluated concentration range.

### *In vivo* tolerability assessment of Qct-Zn nanoparticles

**Figure 9:**
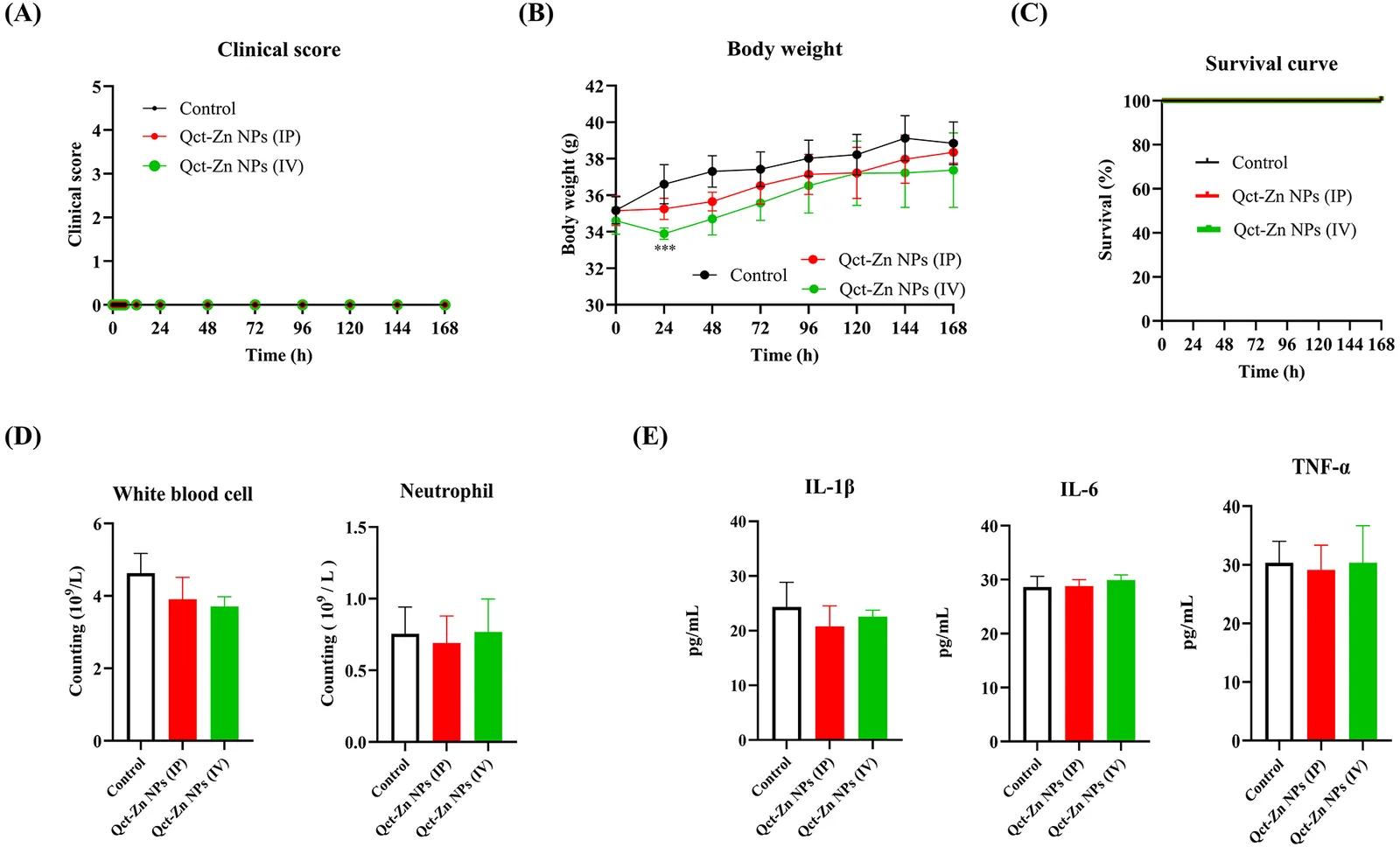
*In vivo* safety of Qct-Zn NPs. **(A)** Acute toxicity of Qct-Zn NPs was evaluated in a mouse model, with 37.5 mg/kg of Qct-Zn NPs per mouse administered via intraperitoneal (IP) or intravenous (IV) routes. Clinical scores were recorded, where “0” denotes healthy status and “5” indicates death. **(B)** Changes in body weight over the study period. At each time point, body weight was compared among the Control, IP, and IV groups using one-way ANOVA followed by Tukey's multiple comparisons test. ***p < 0.001 for IV vs. Control. **(C)** Survival status of mice in each group after injection over the study period. **(D)** White blood cell and neutrophil counts were measured through routine blood tests at the study endpoint. Statistical comparisons among groups were performed using one-way ANOVA, and no statistically significant differences were detected (*P* > 0.05). **(E)** Levels of IL-1β, IL-6, and TNF-α in the blood were quantified using ELISA kits at the study endpoint. Statistical comparisons among groups were performed using one-way ANOVA, and no statistically significant differences were detected (*P* > 0.05).

We further evaluated the preliminary *in vivo* tolerability of Qct-Zn NPs in a murine model. Mice were administered Qct-Zn NPs at 37.5 mg/kg via either intraperitoneal (IP) or intravenous (IV) injection once daily for six consecutive days. Following administration, animals remained active and exhibited no apparent changes in general appearance or behavior during the observation period (Figure 9A).

Body weight monitoring revealed a transient reduction after intravenous administration of Qct-Zn NPs, with a significant decrease observed at 24 h compared with the control group. However, body weight gradually recovered thereafter, and no significant differences among groups were detected from 72 h onward (Figure 9B). At the end of the observation period (168 h), body weights of treated groups were comparable to those of the control group, suggesting that the initial weight reduction did not persist during the observation period (Figure 9B).

After six consecutive administrations, all mice survived throughout the observation period without apparent clinical abnormalities (Figure 9C). Hematological analysis performed on day 7 revealed no significant differences in white blood cell or neutrophil counts among the control, IP, and IV groups (Figure 9D). Similarly, serum levels of inflammatory cytokines, including IL-1β, IL-6, and TNF-α, showed no significant differences among groups (Figure 9E), indicating that repeated administration of Qct-Zn NPs did not cause significant alterations in the measured hematological and inflammatory parameters.

Collectively, these findings indicate that Qct-Zn NPs exhibited favorable *in vivo* tolerability within the evaluated dose, administration schedule, and observation period.

### *In vivo* therapeutic efficacy of Qct-Zn nanoparticles in a murine systemic MRSA infection model

To evaluate the *in vivo* antibacterial efficacy of Qct-Zn NPs, a murine systemic MRSA infection model was established. Different bacterial inocula were initially evaluated to determine an appropriate infection dose. Intraperitoneal administration of 1 × 10^7^ CFU of *S. aureus* 9-11 caused mild clinical symptoms without mortality, whereas 5 × 10^7^ CFU resulted in partial mortality. In contrast, infection with 1 × 10^8^ CFU induced severe clinical manifestations and complete mortality within 36 h (Supplementary Figure 3). Therefore, 1 × 10^8^ CFU per mouse was selected for subsequent therapeutic evaluation.

**Figure 10:**
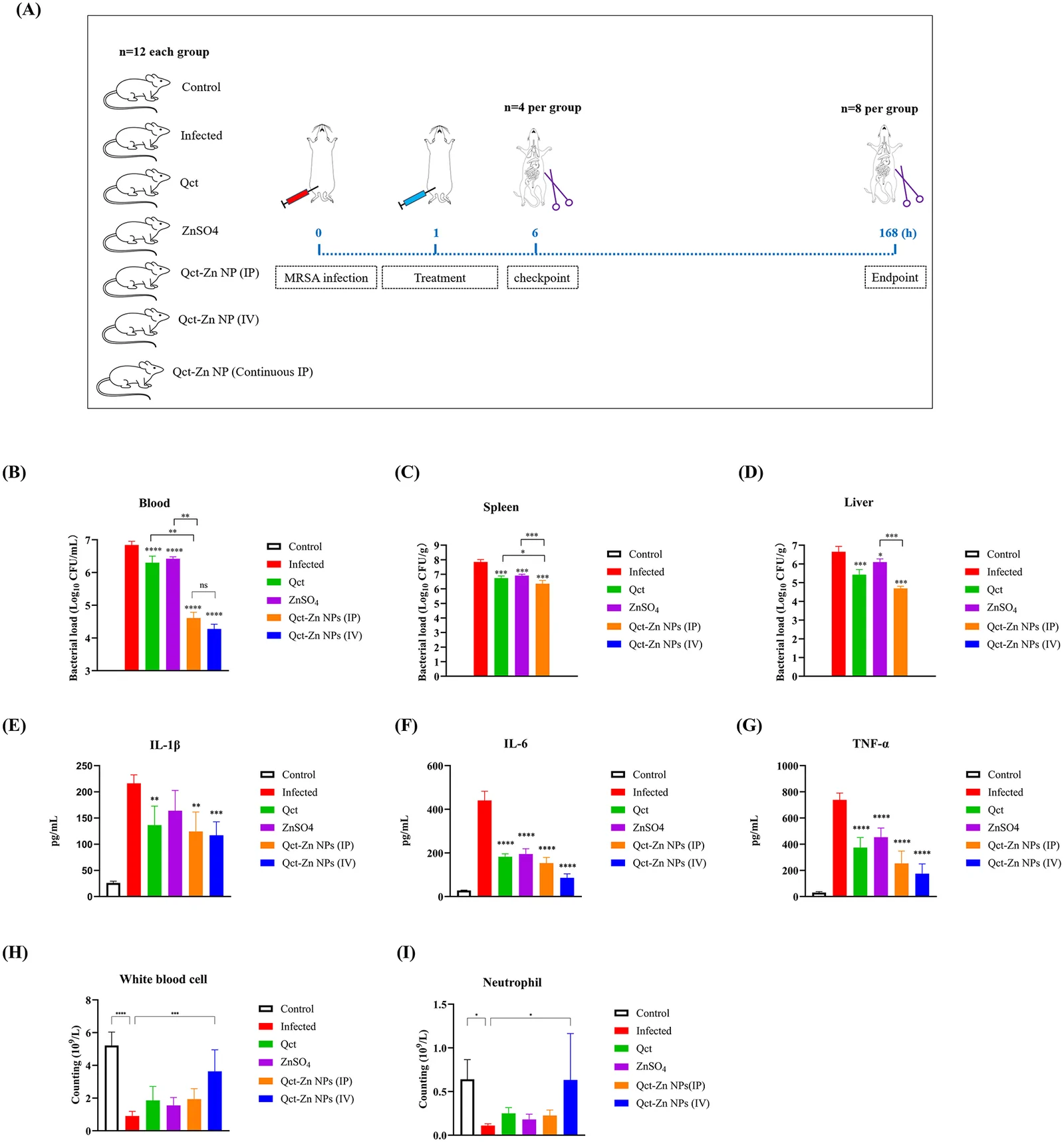
*In vivo* therapeutic efficacy of Qct-Zn NPs in a murine MRSA bacteremia model. **(A)** Schematic illustration of the experimental design for evaluating the therapeutic efficacy of Qct-Zn NPs against MRSA 9-11-induced bacteremia. Mice were intraperitoneally infected with MRSA and treated 1 h after infection with quercetin (Qct), ZnSO_4_, or Qct-Zn NPs administered by intraperitoneal (IP) or intravenous (IV) injection. **(B–D)** Bacterial burdens in the blood **(B)**, spleen **(C)**, and liver **(D)** of infected mice at 6 h after treatment. Bacterial loads were determined by serial dilution and colony counting. In some organs from the IV Qct-Zn NP-treated group, no viable bacteria were detected within the detection limit of the assay. **(E–G)** Serum levels of inflammatory cytokines, including IL-1β **(E)**, IL-6 **(F)**, and TNF-α **(G)**, measured at 6 h after treatment. **(H, I)** White blood cell **(H)** and neutrophil **(I)** counts measured after treatment. Data are presented as mean ± SD. Statistical analyses were performed using one-way ANOVA followed by multiple comparisons tests. Asterisks indicate statistical comparisons between each treatment group and the infected group. Additional brackets indicate comparisons between Qct-Zn NP-treated groups and the corresponding free component groups (Qct or ZnSO_4_). *p < 0.05, **p < 0.01, ***p < 0.001, ****p < 0.0001.

Treatment was initiated at 1 h post-infection. Mice were administered quercetin (7.5 mg/kg), ZnSO_4_ (3.75 mg/kg), or Qct-Zn NPs (7.5 mg/kg) through intraperitoneal or intravenous injection, while an additional group received repeated intraperitoneal administration of Qct-Zn NPs once daily for six consecutive days (Figure 10A). At 6 h POI, bacterial burdens in blood, spleen, and liver were quantified by colony counting.

Compared with untreated infected mice, all treatment groups showed significant reductions in bacterial loads in the examined tissues (Figures 10B–D). Qct-Zn NPs exhibited stronger antibacterial activity than free quercetin or ZnSO_4_, indicating enhanced antibacterial efficacy following nanoparticle assembly. In blood samples, Qct-Zn NP treatment resulted in more than 2-log reduction of bacterial burden, with intravenous administration showing a greater reduction than intraperitoneal administration (Figure 10B). In spleen and liver tissues, intravenous administration reduced bacterial counts to or below the assay detection limit, whereas residual bacteria remained detectable in some tissues following intraperitoneal treatment (Figures 10C, D). These findings indicate that different administration routes influenced bacterial reduction in the evaluated tissues under the tested conditions.

Inflammatory responses were further evaluated following treatment. Compared with infected controls, Qct-Zn NP-treated mice exhibited reduced levels of inflammatory cytokines, including IL-1β, IL-6, and TNF-α, together with partial normalization of white blood cell and neutrophil counts (Figures 10E–I). Histopathological examination further revealed that Qct-Zn NP treatment alleviated infection-associated tissue damage, including hepatic degeneration, splenic congestion, and renal pathological changes observed in untreated infected mice (Supplementary Figure 4). These results demonstrate that Qct-Zn NPs reduced bacterial burden and were associated with attenuation of infection-associated inflammatory responses and tissue injury.

### Therapeutic efficacy of delayed Qct-Zn nanoparticle treatment in a murine systemic MRSA infection model

To further evaluate the therapeutic efficacy of delayed Qct-Zn NP administration, a delayed-treatment experiment was performed using the murine systemic MRSA infection model. Mice were treated with Qct-Zn NPs either 1 h or 6 h after infection, while vancomycin administered at 6 h post-infection was included as a positive control. Clinical signs and body weight changes were monitored throughout the 7-day observation period.

**Figure 11:**
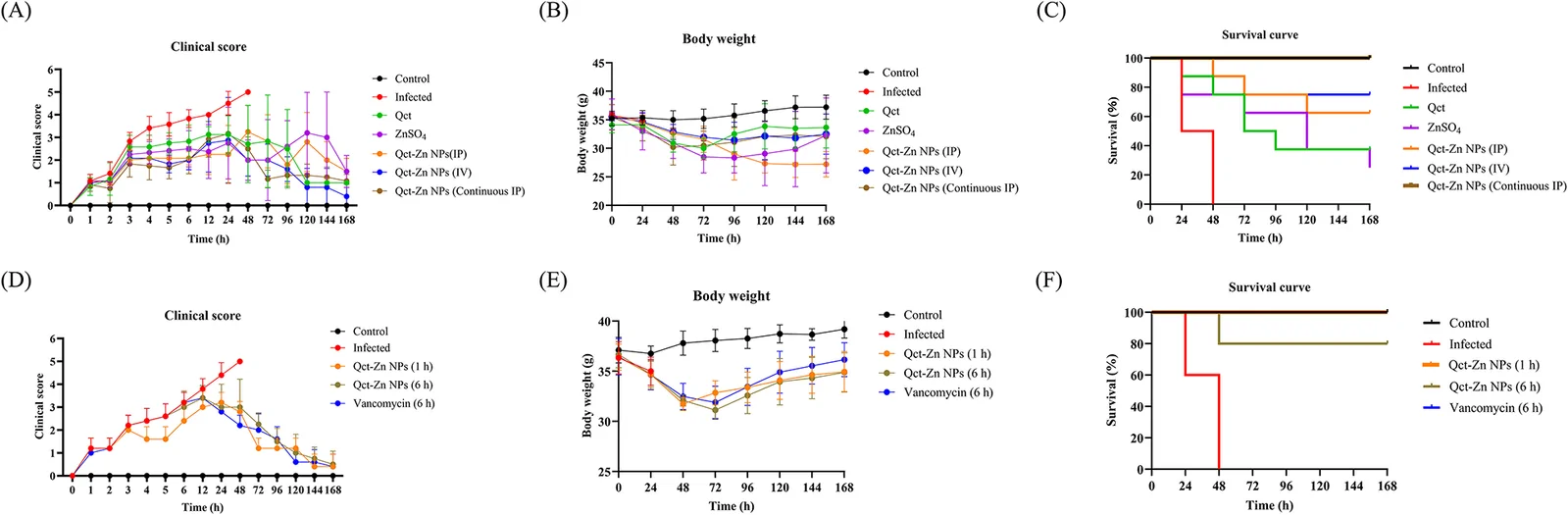
Therapeutic efficacy of Qct-Zn NPs in murine MRSA bacteremia models. **(A–C)** Clinical scores, body weight changes, and survival curves of mice treated with PBS, Qct, ZnSO_4_, single-dose Qct-Zn NPs administered intraperitoneally (IP) or intravenously (IV), or repeated IP administration of Qct-Zn NPs once daily for six consecutive days following therapeutic intervention initiated 1 h post-infection. **(D–F)** Clinical scores, body weight changes and survival curves in a delayed-treatment MRSA bacteremia model. Mice were infected intraperitoneally with 1 × 10^8^ CFU of MRSA and treated with Qct-Zn NPs (7.5 mg/kg, IP) at either 1 h or 6 h post-infection. Vancomycin (30 mg/kg, IP) administered 6 h post-infection served as a standard-of-care comparator. Treatments were given once daily for six consecutive days. PBS-treated mice served as infected controls. Data are presented as mean ± SD. Survival differences were analyzed using the log-rank test.

PBS-treated infected mice exhibited progressive deterioration, as indicated by increased clinical scores during the observation period (Figure 11D). In contrast, Qct-Zn NP treatment alleviated infection-associated clinical symptoms. Early administration at 1 h post-infection resulted in more rapid improvement, whereas delayed administration at 6 h post-infection still showed beneficial effects compared with the infected control group. Consistently, PBS-treated mice exhibited continuous body weight loss, while Qct-Zn NP-treated groups showed reduced weight loss followed by gradual recovery during the observation period (Figure 11E).

Survival analysis further demonstrated improved outcomes following Qct-Zn NP treatment (Figure 11F). All PBS-treated mice succumbed to infection within 48 h, whereas Qct-Zn NP administration improved survival throughout the 7-day observation period. Early Qct-Zn NP treatment (1 h post-infection) resulted in complete survival under the tested conditions, while delayed administration at 6 h post-infection maintained a high survival rate. Vancomycin treatment initiated at 6 h post-infection also improved survival compared with the untreated group.

Taken together, these findings indicate that Qct-Zn NPs retained antibacterial efficacy when administration was delayed after infection onset and improved clinical outcomes in the murine systemic MRSA infection model under the tested experimental conditions.

## Discussion

In this study, we developed quercetin-zinc nanoparticles (Qct-Zn NPs) through a coordination-driven self-assembly strategy and evaluated their antibacterial activity against MRSA. Rather than introducing a fundamentally new material system, this work provides a simple and biocompatible approach to enhancing the antibacterial performance of a natural flavonoid through metal ion coordination. Metal-polyphenol coordination has been widely explored for constructing functional nanomaterials (Ejima et al., 2013; Wan et al., 2025). However, the biomedical translation of quercetin-zinc coordination assemblies, particularly their antibacterial, anti-biofilm, mechanistic, and *in vivo* therapeutic evaluation against multidrug-resistant pathogens, remains insufficiently characterized. In this context, the primary contribution of the present study is not the introduction of an entirely new quercetin-metal nanomaterial, but rather the comprehensive evaluation of a Qct-Zn NP system integrating antibacterial activity, biofilm-associated effects, preliminary biosafety assessment, and therapeutic efficacy against multidrug-resistant *S. aureus*.

Microscopy analyses (SEM, TEM, and CLSM) suggested that Qct-Zn NPs compromise bacterial membrane integrity, resulting in morphological disruption and increased membrane permeability. However, these ultrastructural observations should be interpreted as cellular alterations associated with Qct-Zn NP treatment rather than direct evidence defining the primary antibacterial mechanism. In the present study, Qct-Zn NP treatment also induced marked intracellular ATP depletion accompanied by elevated ROS production in MRSA. These changes indicate altered cellular physiological states following Qct-Zn NP exposure. However, whether they represent primary antibacterial events or downstream consequences of bacterial injury requires further investigation. Interestingly, unlike Vancomycin, Qct-Zn NPs did not cause substantial extracellular ATP leakage, despite profound reductions in intracellular ATP levels. This difference suggests that Qct-Zn NPs and Vancomycin may affect bacterial cellular physiology through distinct patterns of cellular responses. These findings imply that bacterial killing by Qct-Zn NPs may involve metabolic collapse and oxidative stress rather than extensive membrane lysis alone. Previous studies have shown that quercetin can interfere with DNA gyrase activity and energy metabolism, while Zn^2+^ ions disrupt membrane integrity and enzyme function (Qi et al., 2022; Wei et al., 2022; Vatansever et al., 2013). In addition, both components have been associated with oxidative stress responses in bacteria (Ejima et al., 2017). Therefore, the antibacterial activity of Qct-Zn NPs likely results from multiple concurrent mechanisms involving membrane perturbation, oxidative stress, and disruption of bacterial energy homeostasis. Considering that the current study evaluated cellular responses at defined experimental endpoints, the observed membrane-associated alterations, ROS-associated fluorescence changes, and ATP reduction are best interpreted as phenotypic responses associated with antibacterial activity. Future studies combining genetic approaches, ROS scavenging strategies, alternative ROS detection methods, and temporal analyses will be required to further dissect whether ROS-associated responses contribute directly to Qct-Zn NP-mediated bacterial inhibition or represent downstream consequences of bacterial injury.

Importantly, Qct-Zn NPs demonstrated antibacterial efficacy in a murine systemic MRSA infection model, reducing bacterial burdens in blood and major organs, attenuating infection-associated inflammatory responses, and improving survival outcomes under the tested conditions. Compared with free quercetin or ZnSO_4_, Qct-Zn NPs exhibited increased antibacterial activity, suggesting that nanoparticle formulation may improve the antibacterial performance of quercetin and Zn-containing components. Although free component and physical mixture controls were included, the relative contribution of nanoparticle architecture, component availability, and Zn^2+^/quercetin release behavior cannot be fully distinguished based on the current experiments. Further studies are required to clarify the physicochemical determinants underlying the enhanced antibacterial activity. Qct-Zn NPs also maintained antibacterial activity when treatment initiation was delayed to 6 h post-infection within the experimental timeframe, resulting in approximately 80% survival. Although vancomycin provided complete protection under the tested conditions, this comparison was performed within the specific experimental design and should not be interpreted as a direct evaluation of clinical therapeutic equivalence. Beyond antibacterial efficacy, Qct-Zn NPs exhibited low cytotoxicity toward mammalian cells and minimal hemolytic activity under the evaluated conditions. Together with the *in vivo* tolerability assessment, these findings support the preliminary biocompatibility of Qct-Zn NPs within the tested experimental settings.

Despite these encouraging findings, several limitations should be acknowledged. First, although the formation of Qct-Zn nanoscale assemblies was supported by multiple physicochemical analyses, including TEM, UV-VIS, FTIR, DLS, zeta potential, and XPS, the detailed coordination architecture remains to be further clarified. In particular, coordination stoichiometry, ion release behavior, and the relationship between nanoparticle composition and biological activity require additional investigation. Moreover, because XPS is surface-sensitive, the RSF-corrected elemental composition should be interpreted as surface composition rather than the overall Zn-to-quercetin stoichiometry of the assemblies. Although quantitative determination of total Zn content and quercetin incorporation efficiency would provide additional information regarding nanoparticle composition, these analyses were beyond the scope of the current study, which primarily focused on physicochemical characterization and biological evaluation of the assembled system. Second, although membrane-associated alterations, changes in ATP levels, and ROS-associated signals were observed after Qct-Zn NP treatment, the relative contribution of these cellular responses and the roles of intact nanoparticles versus released quercetin or Zn^2+^ require further investigation. In addition, the increased antibacterial activity of Qct-Zn NPs compared with individual components should be interpreted as enhanced combined activity rather than definitive synergism, as formal synergy analysis was not performed. Third, although Qct-Zn NPs reduced biofilm formation and decreased viable bacteria within established biofilms, the underlying mechanisms responsible for these effects remain to be elucidated. Furthermore, the present biofilm experiments were primarily designed to evaluate phenotypic changes, including reduced biofilm biomass and decreased viability of biofilm-associated bacteria, rather than to determine whether Qct-Zn NPs directly interfere with biofilm-specific regulatory pathways. Future studies using additional clinical isolates, sub-inhibitory concentrations, and molecular approaches will be required to further clarify the generalizability and mechanisms of their potential effects on biofilm development. Finally, although *in vivo* therapeutic efficacy was demonstrated in a murine systemic MRSA infection model, further studies involving pharmacokinetics, biodistribution, long-term safety evaluation, and additional clinical isolates will be required to better understand the translational potential of Qct-Zn NPs.

Overall, this study demonstrates that coordination-associated assembly of natural polyphenols and biocompatible metal ions provides a potential strategy for developing antimicrobial nanomaterials. Further investigations into mechanism, pharmacological optimization, and *in vivo* behavior will help advance the future development of Qct-Zn NPs.

## Conclusion

In summary, Qct-Zn NPs formed through coordination-associated self-assembly of quercetin and Zn^2+^ exhibited enhanced antibacterial and anti-biofilm activities against multiple MRSA clinical isolates. Mechanistic investigations revealed membrane-associated alterations, ATP changes, and ROS-associated responses following Qct-Zn NP treatment, suggesting their potential involvement in antibacterial activity. *In vivo* studies demonstrated that Qct-Zn NPs reduced bacterial burdens, attenuated inflammatory responses, and improved survival outcomes in murine systemic MRSA infection models. Notably, Qct-Zn NPs retained antibacterial efficacy when treatment was initiated 6 h post-infection, indicating their activity under delayed-treatment experimental conditions. Together with their favorable preliminary biocompatibility profile, these findings suggest that coordination-driven assembly of natural flavonoids and biocompatible metal ions represents a potential approach for developing antimicrobial materials against MRSA infections. Further investigations addressing pharmacokinetics, ion release behavior, dose optimization, and long-term safety will be required to evaluate the translational potential of this platform.

## Data Availability

The original contributions presented in the study are included in the article/Supplementary material, further inquiries can be directed to the corresponding authors.
